# Apocarotenoid signaling regulates meristem activity and shapes shoot and root lateral organ formation in Arabidopsis

**DOI:** 10.1093/plphys/kiaf414

**Published:** 2025-09-24

**Authors:** Julio Sierra, Lina Escobar-Tovar, Selene Napsucialy-Mendivil, Omar Oltehua-López, Kenny Alejandra Agreda-Laguna, Joseph G Dubrovsky, Ryan P McQuinn, Patricia León

**Affiliations:** Departamento de Biología Molecular de Plantas, Instituto de Biotecnología, Universidad Nacional Autónoma de México, Cuernavaca, Morelos 62210, Mexico; Departamento de Biología Molecular de Plantas, Instituto de Biotecnología, Universidad Nacional Autónoma de México, Cuernavaca, Morelos 62210, Mexico; Departamento de Biología Molecular de Plantas, Instituto de Biotecnología, Universidad Nacional Autónoma de México, Cuernavaca, Morelos 62210, Mexico; Departamento de Biología Molecular de Plantas, Instituto de Biotecnología, Universidad Nacional Autónoma de México, Cuernavaca, Morelos 62210, Mexico; Departamento de Biología Molecular de Plantas, Instituto de Biotecnología, Universidad Nacional Autónoma de México, Cuernavaca, Morelos 62210, Mexico; Departamento de Biología Molecular de Plantas, Instituto de Biotecnología, Universidad Nacional Autónoma de México, Cuernavaca, Morelos 62210, Mexico; School of Science, Western Sydney University, Penrith, NSW 2750, Australia; Departamento de Biología Molecular de Plantas, Instituto de Biotecnología, Universidad Nacional Autónoma de México, Cuernavaca, Morelos 62210, Mexico

## Abstract

Plastids synthesize signals crucial for plant development, including carotenoid-derived molecules with hormonal and retrograde signaling functions that regulate nuclear gene expression, which is an emerging research area. Here we investigate the function of the plastid-derived apocarotenoid signal 1 (ACS1), whose accumulation disrupts plastid biogenesis, affects lateral organ formation, and compromises apical meristem maintenance. By modulating ACS1 levels in Arabidopsis (*Arabidopsis thaliana*) through different light conditions, we show its reversible and dynamic role in leaf and root development. Notably, the characteristic morphological defects of ACS1-accumulating mutants revert under conditions that limit its synthesis, even several days post-germination. This indicates that ACS1 does not cause irreversible damage but rather acts as a signal produced under specific tissues and conditions that associates with the cell-specific expression of its biosynthetic enzymes. Transcriptomic analysis of ACS1-accumulating mutants shows a critical developmental window during which ACS1 affects the expression of numerous plastid-housekeeping genes, correlating with an early block in plastid biogenesis after DNA replication and before transcriptional activation. This disruption affects chloroplast biogenesis and amyloplast starch accumulation. ACS1 accumulation also alters the expression of key developmental regulators, including genes involved in auxin signaling and transport, leading to compromised meristem maintenance and leaf expansion. Beyond photosynthetic tissues, ACS1 also disrupts root apical meristem organization, notably altering columella cell patterning and gravitropic responses. Overall, our findings establish ACS1 as a dynamic conditionally active plastid-derived signal that modulates plastid differentiation, meristem activity, and lateral organ development, underscoring the broader role of *cis*-carotenoid-derived signals in coordinating plastid function with plant growth and development.

## Teaser

Plastid-derived apocarotenoid ACS1 dynamically modulates plastid biogenesis, meristem maintenance, and organ growth, linking plastid-derived signals to coordinate shoot and root development.

## Introduction

Plant fitness and homeostasis largely depend on precise regulation of organ development, which involves continuous crosstalk with organelles, cellular processes and environmental signals. Plant organogenesis is highly flexible, allowing organs to adjust growth and shape for optimal function under changing environmental conditions (Efroni et al. 2010). Primary growth is maintained by 2 meristems: the shoot apical meristem (SAM) and the root apical meristem (RAM) (Stahl and Simon 2010; Lee and Clark 2015), where stem cell niche balances between self-renewal and differentiation for the development of new organs (Aichinger et al. 2012).

SAM and RAM homeostasis is regulated by internal cues (hormones and nutrients) and environmental signals, including light and (a)biotic stresses (Zeng et al. 2024). SAM maintenance depends on a feedback loop between the WUSCHEL (WUS) transcription factor (TF) and the CLAVATA (CLV) pathway (Gaillochet and Lohmann 2015), balancing division and differentiation via regulation of key genes (Schoof et al. 2000; Carles and Fletcher 2003). The RAM stem cell niche consists of quiescent cells (QC) surrounded by multipotent stem cells that form the proliferation domain (Perilli et al. 2012). TFs like WUSCHEL-RELATED HOMEOBOX 5 (WOX5) regulate cell division rates and niche maintenance (Iyer-Pascuzzi and Benfey 2009). The CLAVATA3/EMBRYO SURROUNDING REGION (CLE)-WOX5 module maintains the niche and regulates root cell differentiation (Stahl et al. 2009).

Hormonal cues, including auxin, cytokinin and abscisic acid (ABA), also regulate SAM and RAM maintenance and organogenesis (Zhao et al. 2010; Pacifici et al. 2015). Auxins play crucial roles for meristem homeostasis and lateral organ formation, largely through establishing an auxin-maxima via PIN proteins (Grieneisen et al. 2007; Wang and Jiao 2018) and coordinating leaf development (Burian et al. 2022). In roots, an auxin gradient from the tip to the differentiation zone is critical for meristem maintenance, differentiation (Roychoudhry and Kepinski 2022), and gravitropic responses (Takahashi et al. 2021). Environmental and nutritional factors, including light, nutrient status, and reactive oxygen species (ROS), further regulate SAM and RAM maintenance and activity, influencing TFs such as WUS or AUXIN RESPONSE FACTORS (Zeng et al. 2024), crucial for developmental plasticity and adaptation.

Plastids act as metabolic hubs and environmental sensors, generating regulatory signals that influence meristem organization and function (Chan et al. 2016). In the SAM plastid differentiation is dynamic: proplastids in the central zone either differentiate or revert to an undifferentiated state at the periphery, depending on the cell layer (Charuvi et al. 2012). Plastid biogenesis produces molecules essential for meristem activity and organ development and is coordinated with organ development, as shown by the proplastid to chloroplast transition during leaf development (Andriankaja et al. 2012; Charuvi et al. 2012; Loudya et al. 2024).

Plastids communicate their developmental (biogenic) and functional (operational) status via retrograde signals that regulate thousands of nuclear genes linked to plastid function, cell activity and development (Aluru et al. 2009). They are synthesized during plastid homeostasis and biogenesis and originate from distinct sources (Chan et al. 2016; de Souza et al. 2017; Pfannschmidt et al. 2020; Richter et al. 2023). Retrograde signals influence various developmental programs, including leaf and root formation (Asano et al. 2004; Andriankaja et al. 2012; Avendaño-Vazquez et al. 2014; Van Norman et al. 2014; D'Alessandro et al. 2019). Recent studies in maize and rice indicate that plastid biogenesis occurs in discrete steps, each generating retrograde signals that can either positively or negatively regulate nuclear gene expression (Loudya et al. 2021, 2024; Kendrick et al. 2022). However, the precise nature and associated signaling pathways of most of these signals remain poorly defined.

Apocarotenoids are an emerging class of potential retrograde signals, derived from carotenoid catabolism (Moreno et al. 2021; Sierra et al. 2022). Plants synthesize a variety of carotenoids, which serve as precursors for apocarotenoids. The carotenoid biosynthetic pathway begins with phytoene, which undergoes desaturation and isomerization reactions catalyzed by phytoene (PDS) and zeta-carotene (ZDS) desaturases, and the isomerases ζ-carotene isomerase (Z-ISO) and carotene *cis*-*trans* isomerase (CRTISO), ultimately producing *all-*trans-lycopene (Cazzonelli and Pogson 2010). Unlike bacteria, which utilize a single multifunctional enzyme to convert phytoene to *all-trans*-lycopene phytoene dehydrogenase (CRTI), plants synthesize a variety of poly-*cis* intermediates through these 6 desaturation and isomerization steps (Sandmann 2009). After *all-*trans-lycopene, the pathway diverges producing the cyclic α- and β-carotenoids.

Apocarotenoids are synthesized enzymatically by carotenoid cleavage dioxygenases (CCDs) or non-enzymatically via ROS (McQuinn et al. 2015). Emerging evidence shows that plastid-derived apocarotenoids reprogram nuclear gene expression, modulating developmental, nutritional and stress responses, with some acting as retrograde signals (Moreno et al. 2021; Sierra et al. 2022).

The *CHLOROPLAST BIOGENESIS 5* (*clb5*) and *SPONTANEOUS CELL DEATH 1-2* (*spc1-2*) mutants, defective in ZDS, exhibit early plastid developmental arrest, altered leaf and root morphology and meristem reprograming (Avendaño-Vazquez et al. 2014; McQuinn et al. 2023). These phenotypes result from the over-accumulation of ZDS substrates, followed by their cleavage by the CCD4 enzyme and ROS, to produce an unidentified apocarotenoid referred to as apocarotenoid signal 1 (ACS1). Interestingly, while the absence of CCD4 partially reverts the developmental defects associated with ACS1 overaccumulation, suggesting a more specific role under particular conditions, exposure to low light (<10 *μ*mol m^−2^ s^−1^) results in a near-complete reversion, underscoring a key role for light-dependent ROS contribution to its synthesis (Escobar-Tovar et al. 2021; McQuinn et al. 2023).

ACS1 accumulation triggers extensive nuclear reprograming, particularly of genes encoding plastid proteins like various chlororibosomal proteins, thereby inhibiting plastid translation. The phenotypic similarity in leaf morphology between lincomycin-treated plants and *zds* mutants supports the conclusion that plastid translation deficiency underlies these developmental abnormalities (Escobar-Tovar et al. 2021). Notably, many differentially expressed genes (DEGs) identified in *clb5* are unrelated to plastid function, as observed in other mutants affected in retrograde signaling (Aluru et al. 2009). While the precise chemical structure of ACS1 remains unsolved, functional studies support its role as a potential signal of physiological relevance not only when ectopically expressed. In tomato (*Solanum lycopersicum*), manipulating *ZDS* expression alters ACS1 precursors, leading to changes in inflorescence branching architecture, supporting a conserved physiological role for ACS1 in the development of particular organs such as that of the reproductive meristems (McQuinn et al. 2023).

This study investigates the impact of ACS1 in SAM and RAM maintenance and organ development. By varying light conditions to modulate ACS1 levels, we examined the resulting effects on leaf and root development. Our data demonstrate that ACS1 functions as a plastid-derived signal, directly or indirectly influencing plastid development, meristem activity, and lateral organ maintenance, rather than being a toxic bioproduct. Notably, ACS1 impacts extend beyond photosynthetic tissues, modulating root development. ACS1 overaccumulation leads to columella cells (CC) defects, altered starch accumulation and impaired gravitropic responses. These results underscore the role of *cis*-carotenoid-derived apocarotenoids as key regulators for both shoot and root development.

## Results

### Low light exposure as a method to analyze ACS1 role in development

**Figure 1.**
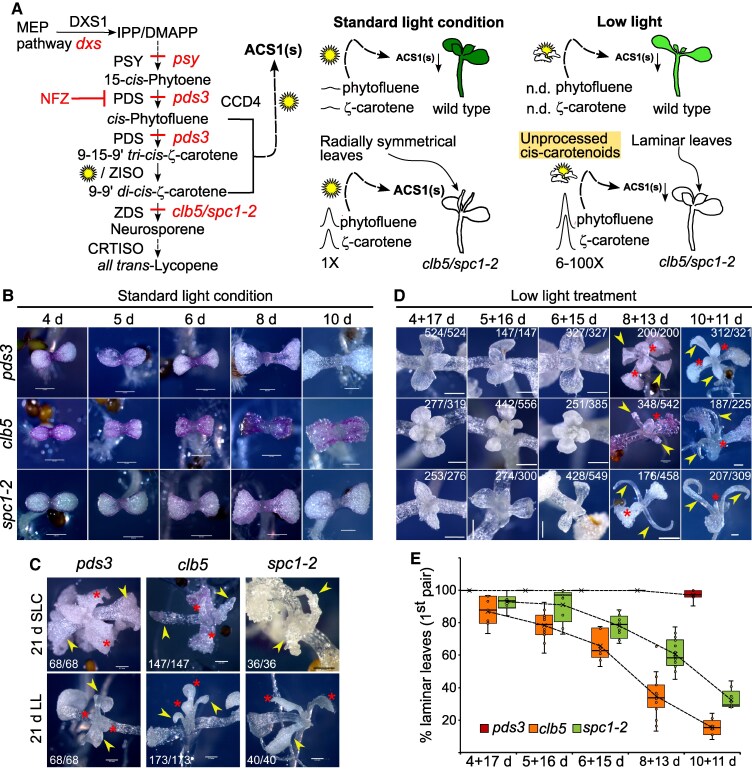
Reversion of developmental leaf defects under low light intensity at different stages of seedling development. **A)** Simplified diagram of the linear-carotenoid biosynthetic pathway, including names of the carotenoid products and the enzymes catalyzing each step (left) are shown. The isopentenyl diphosphate (IPP) and dimethylallyl diphosphate (DMAPP) correspond to precursors for carotenoids, derived from the methyl-D-erythritol 4-phosphate (MEP) pathway. The abbreviations of the enzymes are as follows: deoxy xylulose synthase (DXS), phytoene synthase (PSY), phytoene desaturase (PDS), ζ-carotene isomerase (ZISO), ζ-carotene desaturase (ZDS), and carotene *cis*-*trans* isomerase (CRTISO). Relevant mutants of some of the enzymatic steps are shown and the block for each of them is shown with a line. The diagram indicates that norflurazon (NFZ) blocks carotenoid biosynthesis upstream of ZDS. ZDS substrates, phytofluene, and ζ-carotenes, serve as precursors for ACS1 synthesis via partially redundant cleavage pathways involving carotenoid cleavage dioxygenase 4 (CCD4) and light-dependent ROS. In wild-type (Wt) plants, these precursors remain at very low levels (indicated by a small arrow) during leaf development under both standard (SLC) and low light conditions (Avendaño-Vazquez et al. 2014), suggesting that ACS1 is synthesized transiently and at low levels in specific tissues and/or developmental stages, resulting in normal leaf morphology. In contrast, the *clb5* mutant accumulates high levels of phytofluene and ζ-carotenes due to the defect in the *ZDS* gene promoting ACS1 overproduction in all tissues and developmental stages through enzymatic (CCD4) and non-enzymatic (ROS) cleavage. Under SLC (100 *µ*mol m^−2^ s^−1^), this leads to radial symmetry, whereas under low light (LL; 5 *µ*mol m^−2^ s^−1^), reduced ROS activity limits ACS1 production, restoring laminar leaf morphology. Despite a strong increase in phytofluene (up to ∼100-fold) and ζ-carotenes (6- to ∼100-fold) ACS1 precursors accumulation under LL, inefficient cleavage prevents ACS1 accumulation. Similar responses are observed with the Col allele *spc1-2*. **B)** Representative phenotypes of *pds3*, *clb5*, and *spc1-2* at 4, 5, 6, 8, and 10-d-old (d) albino mutants grown in SLC. **C)** Representative phenotypes of 21-d-old seedlings grown in SLC or LL. Arrows indicate the first pair of leaves and asterisks the second pair of leaves in C and the number in each panel indicates the proportion of individuals with the leaf phenotype indicated by the arrows. **D)** Representative phenotypes of 21-d-old seedlings grown in SLC for the indicated time in each panel, transferred to LL for the indicated times (SLC + LL). The number in each panel represents the individuals analyzed across 5 independent experiments. Arrows point to the first pair of leaves; asterisks mark the second pair. **E)** Quantification of the laminar phenotype in the first pair of leaves in 21-d-old (d) seedlings grown in the indicated SLC + LL treatment shown in panel. The box plot shows the 25th and 75th percentiles of each group’s distribution, with the solid line indicating the median and T-shaped whiskers representing the minimum and maximum values. **D)** Scale bar in B, C, and D = 0.5 mm. In D scale shown in 1 image applies to all images in the panel.

Previous studies have shown that the radially symmetrical primary leaf morphology of the Arabidopsis *clb5* null mutant (Ler ecotype) and its Col-0 *spc1-2* allele reverts to laminar form under low light (LL, 5 *µ*mol m^−2^ s^−1^, 16:8 h light: dark photoperiod) conditions (Escobar-Tovar et al. 2021). A similar reversion is observed in the *clb5* chimeric floral organs under LL (McQuinn et al. 2023). These phenotypic reversions correlate with a 2- to 100-fold over-accumulation of linear-carotenoids, including phytofluene and 19 ζ-carotene isomers, compared to levels under standard light conditions (SLC, 100 *µ*mol m^−2^ s^−^1) and to the undetectable accumulation in Wt seedlings grown under SLC or LL (Fig. 1A) (Avendaño-Vazquez et al. 2014; McQuinn et al. 2023). Together these data support that the altered leaf morphology in *clb5* and *spc1-2* mutants stem from the sustained ACS1 production, via CCD4 activity and non-enzymatic cleavage of ζ-carotene isomers by singlet oxygen (Escobar-Tovar *et al.* 2021; McQuinn *et al.* 2023).

First, the potential dependence of the LL leaf recovery (Escobar-Tovar et al. 2021) on a specific developmental stage or growing conditions was investigated. We examined the leaf morphology in 21-d-old *clb5* and *spc1-2* seedlings when transferred from SLC to LL (SLC + LL) at different times post-germination (Fig. 1, B, D, and E) and compared them to plants grown continuously in SLC (Fig. 1C). The *pds3* mutant serves as a suitable albino control, as it is carotenoid-deficient and lacks ACS1 due to the inhibition of carotenogenesis upstream of ZDS (Escobar-Tovar et al. 2021; McQuinn et al. 2023). Although this mutant may show defects related to the absence of ACS1 in specific conditions or tissues (e.g. floral development), it does not exhibit the developmental abnormalities associated with ACS1's sustained accumulation, such as the fingerlike leaf phenotypes, which are useful to dissect the signaling mechanism of action.

Under SLC 100% of the 21-d-old *clb5* or *spc1-2* primary leaves exhibited radially symmetrical morphology, while 100% of *pds3* leaves developed laminar structure (Fig. 1C). Notably, when *clb5* and *spc1-2* mutants were transferred to LL after 4 or 5 d in SLC, 85% of *clb5* and 90% of *spc1-2* seedlings developed laminar leaves (Fig. 1, D and E). The recovery rate is comparable to that observed in seedlings grown continuously under LL (Fig. 1C) and aligns with previous findings (Escobar-Tovar et al. 2021).

However, when transferred to LL later in development, the recovery rate of the first pair of leaves declined significantly, with only 18% of *clb5* and 30% for *spc1-2* seedlings developing laminar leaves after 10 d (Fig. 1, D and E). This suggests that ACS1 overaccumulation affects leaf development at specific stages. Interestingly, seedlings transferred to LL after 8 or 10 d in SLC developed laminar morphology in the second pair of leaves, while the first pair of leaves remained radial.

Since LL induces full phenotypic reversion, unlike the temporary reversion observed in the absence of CCD4 (McQuinn et al. 2023), it provides a valuable condition for manipulating ACS1 accumulation and further investigating ACS1's function across different tissues, developmental stages, and environmental conditions, as well as its role in plastid biogenesis. The reduction of ACS1 accumulation under low light, as well as the impairment of its biosynthesis in *pds3* or NFZ-treated plants (Fig. 1A), alleviates the fingerlike leaf morphology caused by ectopic ACS1 overaccumulation. This further supports the idea that ACS1 functions in a context-dependent manner, limited to specific conditions and developmental stages.

### Shoot apical meristem maintenance is altered by ACS1

**Figure 2.**
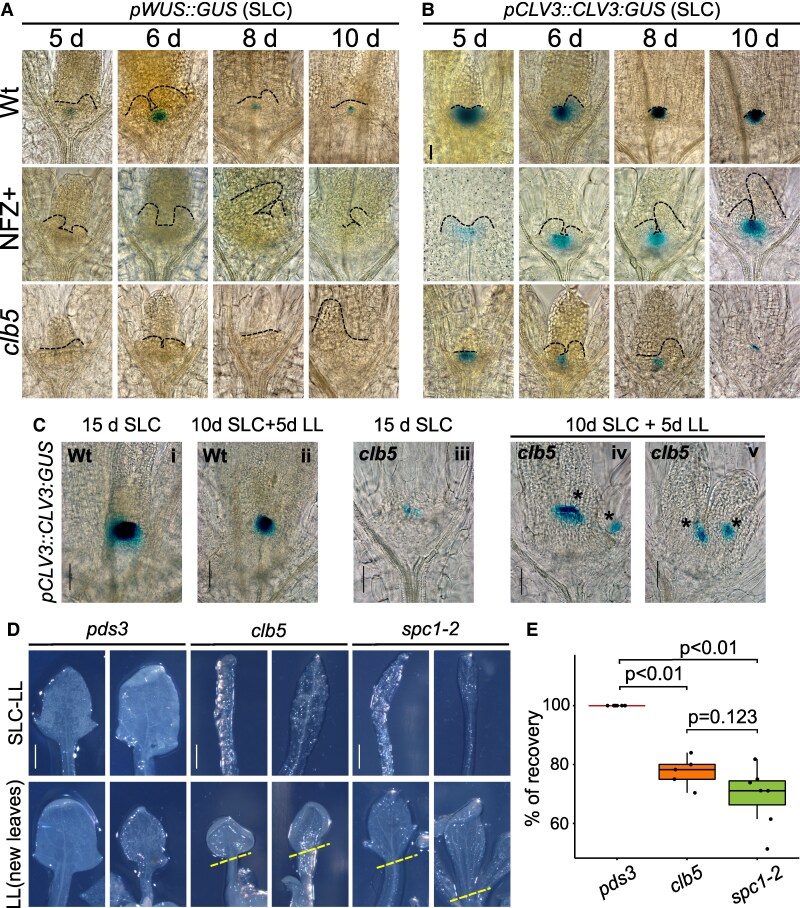
The WUS-CLV3 signaling system is affected in *clb5* causing morphological defects in the SAM that are reverted when ACS1 is reduced. Expression pattern of **A)**  *WUSCHEL* (*pWUS⸬GUS*) and **B)**  *CLAVATA3* (*pCLV3⸬CLV3:GUS*) markers in Ler wild-type (Wt), Ler NFZ-treated (+NFZ) and *clb5* mutant grown in standard light conditions (SLC) during 5, 6, 8, and 10 d. **C)** CLV3 expression pattern in Ler Wt (i) and *clb5* (iii) seedlings grown for 15 d under SLC and Ler Wt (ii) and *clb5* (iv and v) grown 10 d in SLC plus 5 d in LL. **D)** Representative images of *pds3*, *clb5* and *spc1-2* the first pair of leaves developed under standard light conditions for 15 d and transferred to LL for 15 d (SLC-LL) and second pair of leaves developed for 15 d following transfer to LL (LL new leaves). The dashed lines mark the base of the new formed leaves grown under LL. **E)** Percentage of laminar leaves (% recovery) shown in D in *clb5* and *spc1-2* compared to *pds3* (15SLC + 15LL) across 5 independent analyses. The box plot shows the 25th and 75th percentiles of each group’s distribution, with the solid line indicating the median and T-shaped whiskers representing the minimum and maximum values. Total number of pooled individuals was 451 for *pds3*, 190 for *clb5* and 600 for *spc1-2*. Pairwise comparison was done using the Tukey HSD test. Dashed lines in A and B show the SAM architecture and developing leaves boundaries in each background. Ectopic *CLV3* expression is marked with an asterisk (*) in C. Scale bar in A, B, and C, 50 *μ*m (scale shown in 1 image applies to all images in the panel); **D)** 0.5 mm (scale shown in 1 image applies to all images).

Previous studies have shown that under SLC, *clb5* and *spc1-2* mutants produced only 2 primary radial leaves, halting further organ development. In contrast, with 3% sucrose, *clb5* SAM undergoes identity reprograming, producing 3 to 4 terminal organs with floral traits (McQuinn et al. 2023). Given that SAM maintenance affects leaf development and vice versa (Qi et al. 2014), we investigated potential developmental alterations in the SAM of *clb5* and *spc1-2* mutants and their relationship to ACS1 accumulation. To analyze *WUS* and *CLV3* expression patterns as reporters for SAM organization (Somssich et al. 2016; Shimotohno 2022), both *pWUS⸬GUS* and *pCLAVATA3⸬CLAVATA3:GUS* (*CLV3⸬GUS*) reporters (Brand et al. 2002; Bäurle and Laux 2005) were introduced into the *clb5* background and their expression compared to that of wild-type (Wt) Ler and Ler norflurazon (NFZ)-treated plants under SLC and LL conditions as controls. NFZ inhibits the PDS3 enzyme, blocking carotenogenesis and inducing retrograde signals due to photooxidative stress, but without producing finger-leaf morphology, similar to the *pds3* mutant. In Wt seedlings, *WUS* expression was detected in the SAM at all stages analyzed (Fig. 2A). However, in both Wt NFZ-treated and *clb5* seedlings, *WUS* promoter activity was undetectable even after the emergence of the second leaf pair (10-d-old), indicating low *WUS* expression levels. This is likely due to metabolic deficiencies, as previously reported in photosynthesis-deficient seedlings (Pfeiffer et al. 2016). Therefore, the *WUS* reporter was not suitable for assessing meristem status in response to ACS1 accumulation. Notably, the SAM structure in *clb5* seedlings was altered, lacking the typical dome-like shape observed in Wt and NFZ-treated plants (Fig. 2A).

Unlike *WUS*, *CLV3* expression was clearly detected in the SAM of 5-d-old *clb5* seedlings, resembling that of Ler NFZ-treated seedlings and Ler Wt plants, albeit at a reduced level (Fig. 2B). While *CLV3* expression increased throughout seedling development in Wt NFZ-treated plants, it decreased in *clb5,* showing especially low levels in 10-d-old seedlings (Fig. 2B). This pattern suggests ACS1 signal accumulation may disrupt the WUS-CLAVATA pathway, potentially leading to the depletion of the *clb5* stem cell population.

Interestingly, when 10-d-old *clb5* seedlings with low *CLV3* expression (Fig. 2B) were transferred to LL for 5 additional d (Fig. 2Civ and v), *CLV3* levels recovered significantly compared to seedlings grown in SLC for 15 d (Fig. 2Ciii). This recovery highlights the plasticity of the *clb5* and *spc1-2* mutants and the dynamic effect of ACS1. Notably, 20% of these seedlings displayed ectopic *CLV3* expression (Fig. 2Civ and v), not observed in Wt plants under either SLC (Fig. 2Ci) or after transfer to LL (Fig. 2Cii). Given that *CLV3* marks shoot stem cells (Brand et al. 2002), this ectopic expression likely indicates regions acquiring stem cell identity.

To further analyze *clb5*'s stem cell recovery under LL when ACS1 levels decrease, we transferred 15-d-old SLC-grown seedlings, with low *CLV3* expression (Fig. 2Ciii) to LL for an additional 15 d. These *clb5* and *spc1-2* seedlings (15 d SLC + 15 d LL), initially containing 2 fingerlike leaves, continued growing in LL up to 30 d (Supplementary Fig. S1A), producing new leaves (Fig. 2D) with recovered laminar phenotypes (69% and 77%, Fig. 2E), in contrast to seedlings maintained under SLC, which remained developmentally arrested (Fig. 1C). To quantify these differences, we compared the circularity index between the initial fingerlike leaves that developed under SLC and those produced under LL, confirming significant differences (Supplementary Fig. S1B). These findings indicate that ACS1 not only can influence leaf development but also fine-tune SAM activity.

### Gene expression profile in response to ACS1 accumulation highlights its critical role during plastid biogenesis

Given that meristematic defects in *clb5* were not evident during early development (Figs. 1 and 2B) but emerged later, we investigated whether these defects correlate with specific gene expression changes. To do so, we analyzed and compared global transcriptomic (RNA-seq) data from 8- and 18-d-old *clb5* and *pds3* mutant seedlings (Escobar-Tovar et al. 2021). These time points correspond to key developmental stages: seedlings with fully expanded cotyledons and early-developing primary leaves (Stage 1.0 referred to as cotyledon stage), and seedlings with fully expanded primary leaves (Stage 1.02) (Boyes et al. 2001). These stages are ideal for assessing ACS1’s impact during leaf developmental transitions (Andriankaja et al. 2012; Loudya et al. 2021).

**Figure 3.**
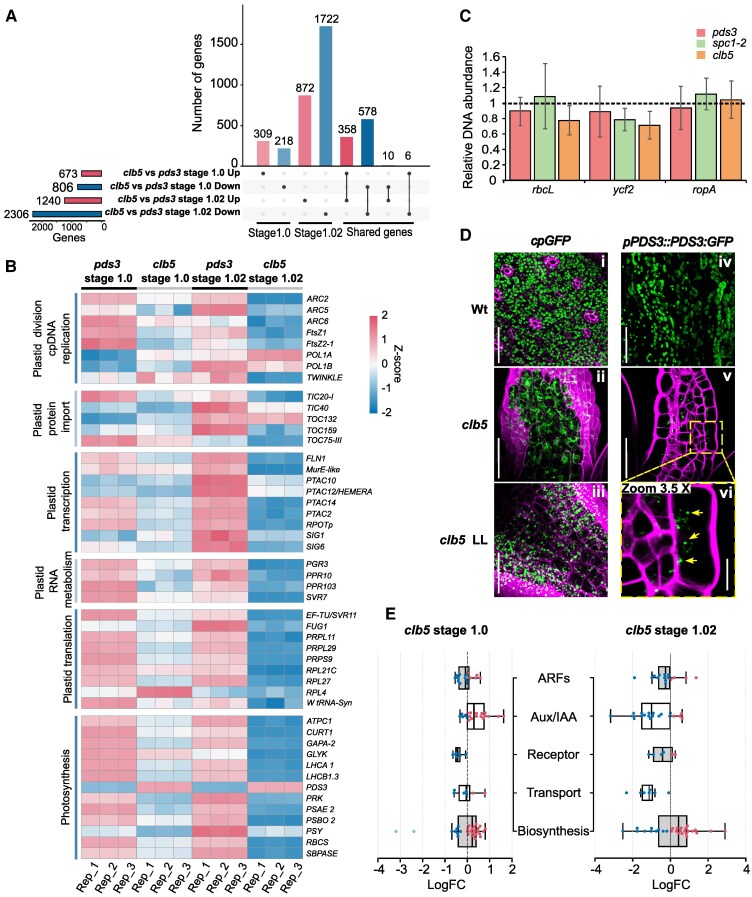
Analysis of differentially expressed genes of *clb5* along development. **A)** Upset plot of DEGs identified at stages 1.0 (cotyledon) and 1.02 (leaf) in the *clb5* mutant compared to *pds3* mutant, used as control. The total number of upregulated or downregulated DEGs for each comparison is shown to the left of the total genes counts. This total corresponds to the sum of condition-specific and shared DEGs (either upregulated in both conditions or exhibiting contrasting responses), as indicated by the black dots beneath the upset plot. **B)** Heatmap of genes encoding proteins located in the chloroplast of *clb5* and *pds3* using a *Z*-score and *P*-Value <0.05 of the DEGs, each condition contains 3 independent replicates represented in each column. **C)** Number of copies of the plastid genome of *pds3*, *spc1-2*, and *clb5* mutants relative to their corresponding Col or Ler Wt taken as 1 (dotted line). Data represent the mean ± standard deviation (SD) of 3 biological independent analyses from a pooled sample of 10 mg of seedlings. Plastid copy number was obtained using 3 plastid-encoded genes normalized against the *HEMA1* and *RPOTp* nuclear genes. **D)** Confocal images the plastid showing GFP localization from the plastid marker *pro35S⸬RBCSctp:GFP* (*cpGFP*) of wild-type (Wt; i) and *clb5* leaves grown under standard light (ii) (100 *µ*mol m^−2^ s^−1^) or low light (iii) conditions (*clb5* LL; 5 *µ*mol m^−2^ s^−1^), and *proPDS3⸬PDS3:FP* marker of Wt (iv) and *clb5* under standard light (v). A magnified view (3.5X) of the dashed box in panel v is shown (vi). Cell membranes were stained with PI. The images are representative of 3 independent biological replicates *n* ≥ 10. Bar 50 and 10 *µ*m for the zoom. **E)** Boxplots of expression levels of genes involved in auxin transport and signaling in *clb5* at 2 developmental stages; the box indicates the 25th and 75th percentile of the distribution of each gene group and the solid line indicates the median value, the T-shaped lines are the maximum and minimum LogFC values; each dot represents a gene.

In Stage 1.0 *clb5* seedlings, we identified 1,479 differentially expressed genes (DEGs) (+/−1.5 log_2_FC; 673-upregulated and 806-downregulated) compared to *pds3,* whereas the Stage 1.02 *clb5* seedlings displayed over 3,500 DEGs (Fig. 3A and Supplementary Table S1). This indicates that ACS1-mediated transcriptional regulation becomes increasingly significant at later stages. Although many DEGs from stage 1.0 persisted at Stage 1.02 (53% upregulated and 71% downregulated genes), both the number of DEGs and fold changes were higher at Stage 1.02 (Fig. 3A). This is unlikely due to stage-specific expression alone, as *pds3* showed minimal expression differences between these 2 stages (Supplementary Fig. S2A). Relative expression levels (*Z*-scores) showed that among the top downregulated DEGs in *clb5* compared to *pds3* were not only the Photosynthesis-Associated Nuclear Genes (PhANGs), but also genes involved in plastid biogenesis, such as transcription and translation (Fig. 3B and Supplementary Table S2). Specifically, we observed reduced expression of subunits of the plastid-encoded polymerase (PEP) subunits, as well as the nuclear-encoded NEP polymerase *RPOTp* (Fig. 3B) (Hedtke et al. 1997; Sriraman et al. 1998).

Interestingly, this significant repression of plastid gene expression, contrasted with stable or even elevated expression of genes related to plastid DNA replication and protein import. For instance, plastid DNA polymerases *POL1A* and *POL1B,* as well as the non-photosynthetic protein import receptor *TOC132*, showed sustained or increased expression in *clb5* relative to *pds3* (Fig. 3B). These trends were validated by RT-qPCR analysis for *spc1-2* allele and its corresponding Wt (Supplementary Fig. S2B).

To support the hypothesis of plastid biogenesis arrest, we quantified plastid DNA copy number by qPCR using 3 single-copy plastid-encoded genes (*rbcL, rpoA*, and *ycf2*) in both *clb5* and *spc1-2* mutants, compared to *pds3* and their respective Wt, using the *HEMA1* and *RpoTp* nuclear genes as reference (Fig. 3C). Despite reduced expression of plastid housekeeping genes in *clb5* and *spc1-2,* plastid DNA copy number remained comparable to those in *pds3* and Wt. These results suggest that plastid DNA replication proceeds normally, but plastid biogenesis is arrested before NEP-mediated transcription. This is consistent with an early developmental block after the increase in plastid DNA copy number, similar to wild-type seedlings (Mullet 1993; Loudya et al. 2024).

To assess plastid import in *clb5*, we used a GFP fusion containing the RBCS transit peptide (*C-cpGFP*) (Wu et al. 2019) and a *pPDS3⸬PDS3:GFP* construct. While PDS3*⸬*GFP localized properly to plastids of both *clb5* and Wt plants, C-cpGFP was retained in the cytoplasm of *clb5* cells, unlike Wt (Fig. 3D). Notably, C-cpGFP plastid import was restored under low light conditions, indicating that the import defect is linked to ACS1 accumulation (Fig. 3D).

These results suggest a selective impairment in the import of photosynthetic proteins such as RBCS, while proteins such as PDS3, expressed in different plastid types remain unaffected, which is in agreement to our transcriptome data and mutants in the plastid translocon (Kikuchi et al. 2013). Together, these results reinforce the model in which ACS1 accumulation halts plastid development at an early biogenic stage, likely after the proliferation phase near to a proplastid stage, and prior to transcriptional activation, although the specific target remains unknown. Taking specifically the chloroplast biogenesis, the timing appears to precede the leaf cell expansion arrest previously reported in NFZ-treated Arabidopsis (Andriankaja et al. 2012; Loudya et al. 2020).

### ACS1-related radial leaf defects are linked to auxin responses

Transcriptome comparison between 1.0 and 1.02 stages also showed significant downregulation at stage 1.02 of auxin transport and signaling genes, including multiple *PIN-FORMED* (*PIN*) and *AUXIN/INDOLE-3-ACETIC ACID* (*IAA*) genes, in *clb5* compared to *pds3* (Fig. 3E). This suggests that disrupted auxin homeostasis may impair meristem maintenance and organogenesis in *clb5*. Meristem maintenance and lateral organ formation involve several hormones, with auxins playing a crucial role. Proper auxin distribution between leaf primordia and SAM is critical for organogenesis (Qi et al. 2014; Somssich et al. 2016).

**Figure 4.**
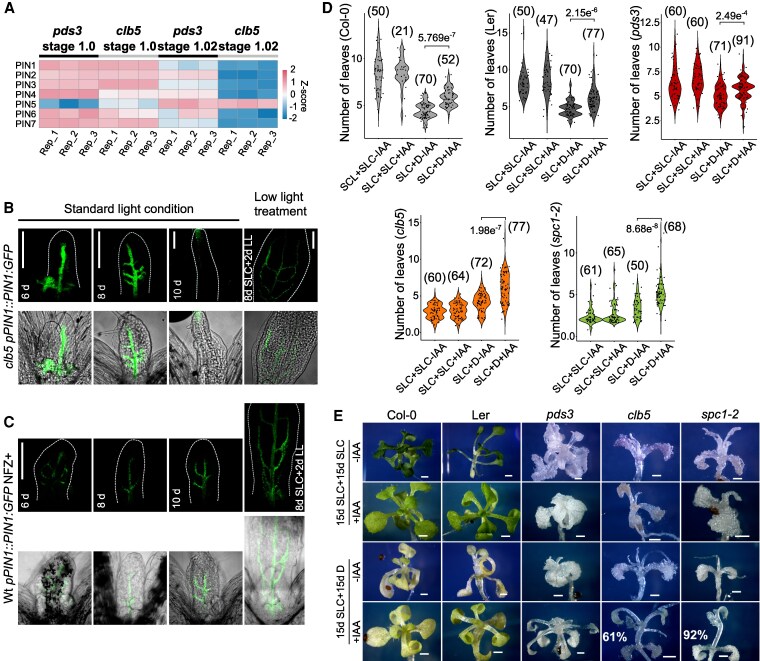
Effect of auxins over the *clb5* and *spc1-2* leaf morphology. **A)** Heat map expression levels of genes involved in auxin transport in *pds3* and *clb5* at 1.0 and 1.02 developmental stages. Confocal images of *proPIN1⸬PIN:GFP* expression in the first pair of leaves in the *clb5* mutant **B)** and Ler Wt NFZ-treated plants **C)** grown in SLC or transfer to LL for 2 d (8d SLC + 2LL). **D)** Number of leaves of 30-d-old Wt (Col-0 and Ler) and carotenoid deficient mutants *pds3*, *clb5*, and *spc1-2* grown in media supplemented with 1% sucrose (+Suc) for 15-d in SLC and transferred to media with 1% sucrose and supplemented with 0.1 *μ*m IAA (+IAA) or without (−IAA) and grow for 15 d in standard light (SLC) or darkness **(D)**. The numbers in parentheses represent the pooled individuals analyzed in 4 independent experiments. Kruskal–Wallis was used for multiple comparisons, and Dunn's test for pairwise comparisons, *P*-values are indicated for relevant comparisons. **E)** Representative phenotypes of the 30-d-old leaves quantified in **(D)**. The % of laminar leaves in *clb5* and *spc1-2* grown in SLC + D + IAA is included. Scale bar for B and C 100 *µ*m and for E 1 mm (scale shown in 1 image applies to all images in the panel).

Previous studies have already shown that sustained ACS1 accumulation in *clb5* leaves under SLC disrupts auxin accumulation and alters PIN1 expression (Avendaño-Vazquez et al. 2014; Escobar-Tovar et al. 2021). Our transcriptomic analysis confirmed the downregulation of several PIN transporters at stage 1.02 (Fig. 4A, Supplementary Table S3). In *clb5* seedlings expressing the *proPIN1:PIN1:GFP* reporter and grown under SLC, vascular expression was detectable at 6 and 8 d (Fig. 4B), but by d 10, expression became almost undetectable, with only minor accumulation at the leaf tip. This pattern is in contrasts to the expression observed in NFZ-treated Wt leaves (Fig. 4C). This pattern correlates with the emergence of the radial symmetry in *clb5* leaves due to leaf elongation without expansion (Fig. 4B). Transferring 8-d-old *clb5* seedlings from SLC to LL for 2 d restored laminar morphology and *proPIN1:PIN1:GFP* expression in 40% of the emerging leaves, indistinguishable from NFZ-treated seedlings (Fig. 4, B and C).

Auxin accumulation, inferred using the *proDR5⸬GUS* reporter, was altered under SLC. Although auxin maxima appeared at the tip of the 10 d-old *clb5* leaves, similar to Wt and NFZ-treated plants, *proDR5⸬GUS* expression was lost by d 15 in *clb5* (Supplementary Fig. S3). Transferring 10-d-old *clb5* seedlings to LL for 5 d, maintained *proDR5⸬GUS* expression (Supplementary Fig. S3). These results link ACS1 accumulation to the misregulation of stage 1.02 auxin-related genes, disrupting auxin distribution and leaf expansion. This underscores ACS1´s dynamic role in regulating leaf development.

Exogenous IAA treatment of 6-d-old *clb5* and *spc1-2* seedlings failed to rescue laminar morphology after 9 d (Supplementary Fig. S4), indicating that ACS1-associated defects are not due to low auxin levels. To test auxin responsiveness at low ACS1 levels, Wt and mutants were grown in darkness with auxins and sugars, a condition that supports leaf development in the dark (Li et al. 2017). Fifteen-day-old Wt, *pds3, clb5*, and *spc1-2* seedlings from SLC were transferred to sucrose media with or without 0.1 mm of IAA and grown for an additional 15 d in the dark or under SLC. A reduction in the number of leaves was observed in dark with sucrose media in all genotypes versus SLC, indicating meristem arrest (Fig. 4D), without significant differences in leaf number or radial leaf morphology recovery in *clb5* and *spc1-2* (Fig. 4, D and E). However, under dark plus sucrose and IAA (SLC + D + IAA), *clb5* and *spc1-2* mutants display a normal auxin response, with an increase in the number of leaves, indicating SAM activation (Fig. 4D). Notably, 61% of newly emerging *clb5* leaves and 92% of *spc1-2* leaves displayed laminar morphology, which was absent in controls without sucrose (Fig. 4E and Supplementary Fig. S5). These findings demonstrate that ACS1-associated leaf defects are reversible upon ACS1 decline and support that *clb5* and *spc1-2* mutants retain auxin responsiveness when ACS1 levels drop.

### ACS1 influences root apical meristem length independently of auxin accumulation

**Figure 5.**
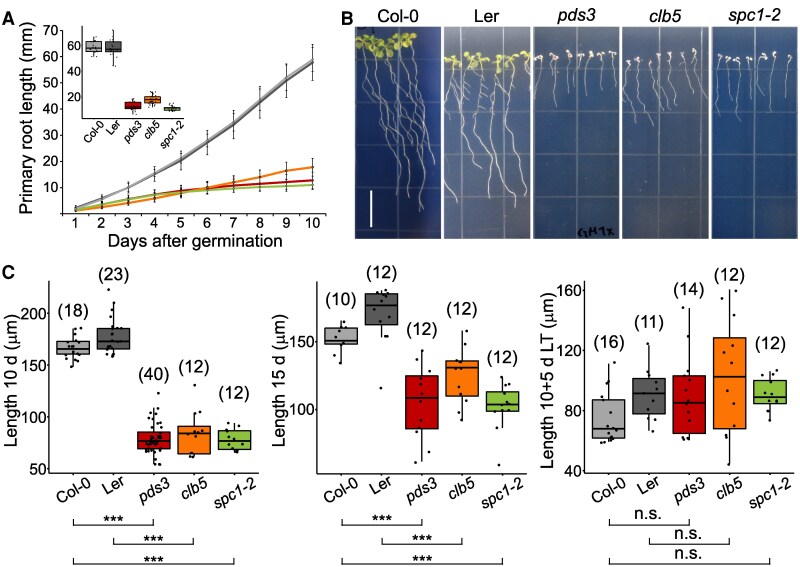
The primary root and RAM sizes are affected in *clb5* mutant. **A)** Primary root growth kinetics of Col-0 and Ler wild-type, *pds3*, *clb5*, and *spc1-2* mutants analyzed over 12 d after stratification. The inset shows the primary root length of the 12-d-old seedlings. The total number of pooled individuals across 2 independent analyses is as follows: 20 for Col-0, 20 for Ler, 22 for *pds3*, 26 for *clb5*, and 25 for *spc1-2*. **B)** Representative morphology of the root system of 12-d-old *pds3*, *clb5*, and *spc1-2* mutants compared with Col and Ler Wt. **C)** Quantification of the RAM length in Wt (Col-0 and Ler), *pds3*, *clb5-1* (*clb5*), and *spc1-2* seedlings grown in SLC for 10 d (10 D), 15 d (15 D), or for 10 d in SLC followed by 5 d in LL (10 + 5 D). For A and C, the box plots show the 25th and 75th percentiles of each group’s distribution, with the solid line indicating the median and T-shaped whiskers representing the minimum and maximum values. Asterisks indicate statistically significant differences with respect to their respective Wt, analyzed by Kruskal–Wallis test (*P* < 0.01) and Dunn's test for pairwise comparisons; n.s., indicates no statistical significance (*P* ≥ 0.01). The numbers in parentheses indicate the total pooled individuals across 2 independent analyses.

Based on previous studies showing altered *proPIN1⸬PIN1:GFP* expression in *clb5* roots (Escobar-Tovar et al. 2021), we investigated the potential influence of ACS1 on root development. Under SLC, primary root growth rates were lower in *clb5*, *spc1-2*, and *pds3* compared to their respective Wt (Ler and Col-0), resulting in shorter and narrower roots (Fig. 5, A and B and Supplementary Fig. S6). However, these defects were not linked to ACS1 accumulation, as *pds3* exhibited similar phenotypes.

Analysis of 10- and 15-d-old *clb5*, *spc1-2*, and *pds3* roots grown under SLC showed shorter RAM lengths compared to Wt. These differences disappeared after 5 additional days in LL, where RAM lengths in Wt and albino mutants were similar (Fig. 5C and Supplementary Fig. S7). The reduced RAM length in both Wt and albino mutants after 5 d of LL exposure confirms their responsiveness to low light (Supplementary Table S4), as previously reported in Wt seedlings (Miotto et al. 2021). Despite similar RAM lengths among the mutants, RAM morphology was altered in *clb5* and *spc1-2* compared to *pds3*, supporting a role for ASC1 in these defects (Supplementary Fig. S7B). Notably RAM morphology recovered after 5 d in LL (Supplementary Fig. S7C).

Additionally, auxin response, based in *proDR5⸬GUS* expression, was similar in *clb5,* Ler and Ler-NFZ-treated plants in the different conditions tested (Supplementary Fig. S8), suggesting an auxin-independent response. Further analysis on 6-d-old *clb5*, *spc1-2, pds3*, and Wt roots for responses to the addition of 0.5 *µ*M ABA, 0.4 *µ*M strigolactone (GR24), and 0.1, 0.2, and 0.4 *µ*M auxin (IAA) over 9 d was carried out (Supplementary Fig. S9). In Wt roots, we did not observe significant differences in the response to ABA and GR24, although some differences were detected in the responses of *pds3*, *clb5*, and *spc1*. Importantly, *clb5* and *spc1-2* roots responded to exogenous auxin similarly to *pds3* and Wt, unlike aerial tissues (Supplementary Figs. S4 and S9). Overall, although there was a statistically significant interaction between the genotypes and hormone treatments, these phenotypes appear to be genotype-specific and not directly related to the presence of ACS1.

### Impact of ACS1 on lateral root primordia initiation and emergence

**Figure 6.**
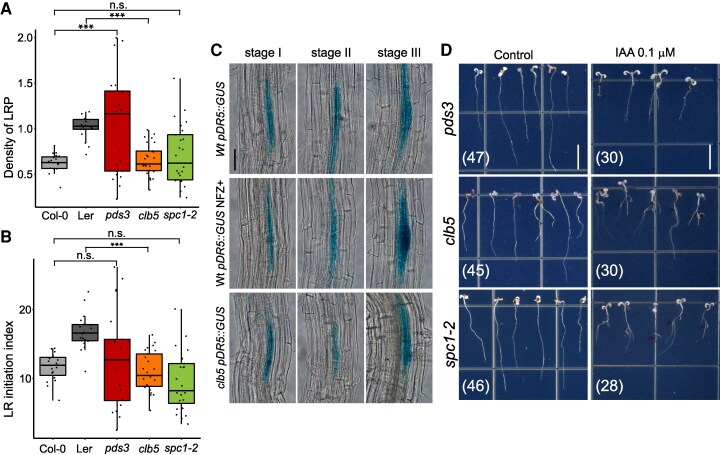
Lateral root development is affected differently in carotenoid deficient mutants. **A)** Lateral root (LR) primordia (LRP) density and **B)** lateral root initiation index was measured in 12-d-old Col and Ler Wt and carotenoid deficient *pds3*, *clb5-1* (*clb5*), and *spc1-2* mutants. For A and B, the box plots show the 25th and 75th percentiles of each group’s distribution, with the solid line indicating the median and T-shaped whiskers representing the minimum and maximum values. Asterisks indicate statistically significant differences with respect to their respective Wt (*P* < 0.01) analyzed by 1-way ANOVA (*P* < 0.01) and Tukey’s HSD test. n.s. indicates no statistical significance (*P* ≥ 0.01). Total number of pooled individuals from 2 independent analyses is as follows: 20 for Col-0, 18 for Ler, 20 for *pds3*, 26 for *clb5*, and 25 for *spc1-2*. **C)** GUS expression pattern of the *proDR5⸬GUS* synthetic auxin marker from Ler (WT), Ler in the presence of norflurazon (NFZ) and *clb5* seedlings grown in standard conditions (SLC, 100 *µ*mol m^−2^ s^−1^) from root initiation stages I, II and III. **D)** LR emergence in *pds3*, *clb5-1* and *spc1-2* mutants in response to 0.1 *μ*m IAA supplementation shown by the asterisks. The numbers in parentheses indicate the total pooled individuals across 5 independent analyses. Scale bar for C 100 *µ*m and for D 1 cm.

Next, we investigated ACS1's role in lateral root (LR) development in *clb5* and *spc1-2* mutants compared to *pds3* and Wt seedlings. All 3 carotenoid-deficient mutants have significantly fewer LRs than Wt (Fig. 5B). In addition, lateral root primordium (LRP) density was assessed. The *clb5* mutant had 50% lower LRP density than Ler Wt (*P* = 0.0029), while *spc1-2* did not differ from Col-0 Wt (*P* = 0.88). Interestingly, *pds3* exhibited an increased LRP density compared to Col-0 (*P* = 0.0022), highlighting different responses between these mutants (Fig. 6A).

To evaluate LR initiation, we calculated the lateral root initiation Index (*l_LRI_*) (Dubrovsky et al. 2009), and normalize by cell length. The *l_LRI_* was lower in *clb5* than Ler (*P* = 0.00019) but similar in *spc1-2* relative to Col (*P* = 0.44). This suggests that ACS1 has an inhibitory effect on LR initiation (Fig. 6B). Further, *proDR5⸬GUS* analysis showed similar auxin maxima during LRP initiation and development in *clb5* primordia to that of Wt and NFZ-treated plants (Fig. 6C). These results indicate that while LR emergence defects are common in all carotenoid-deficient mutants analyzed, ACS1 accumulation specifically inhibits LR initiation in an ecotype-dependent form.

To test whether auxins, ABA and strigolactones could rescue LR development in *clb5* and *spc1-2* mutants, 6-d-old Wt and albino seedlings were treated for 9 d with 0.1 *μ*m IAA, 0.1 *μ*m ABA or 0.5 *μ*m GR24. IAA promoted LR emergence in all mutants (Fig. 6D), indicating exogenous auxin promotes primordia outgrowth. In contrast, ABA or GR24 treatments had no effect in LR emergence, (Supplementary Fig. S10). Together these results indicate that, although ACS1 accumulation inhibits LR initiation and emergence, mutant roots retain responsiveness to exogenous auxin.

### ACS1 accumulation causes defects in RAM and columella morphology

**Figure 7.**
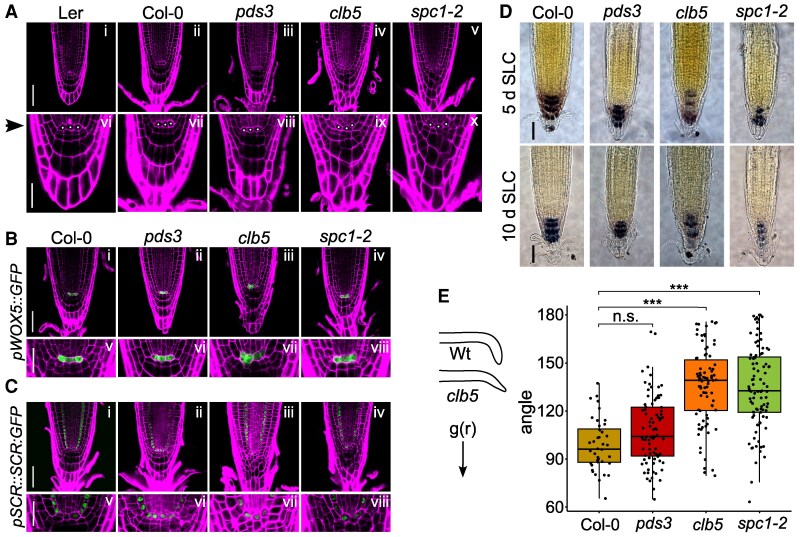
Accumulation of ACS1 affects RAM and QC structure and functionality. **A)** Representative confocal images of root tip morphology from 10-d-old Ler (i) and Col wild type (Wt, ii), *pds3* (iii), *clb5* (iv), and *spc1-2* (v) seedlings, showing morphological defects in the *clb5-1* and *spc1-2* RAMs. Images below show magnified views of the corresponding RAM regions highlighting the QC and columella regions (vi to x). Asterisks in A mark the QC cells, also marked by the left arrow. Expression patterns of *proWOX5⸬GFP*  **B)** and *proSCR⸬SCR:GFP*  **C)** in 10-d-old Col Wt (Bi, Ci), *pds3* (Bii, Cii), *clb5* (Biii, Ciii), and *spc1-2* (Biv and Civ). Images below show magnified views of the corresponding RAMs highlighting the QC and columella initials (Bv to Bviii and Cv to Cviii). Samples were stained with PI for proper visualization. The images are representative of 3 independent biological replicates (*n* ≤ 10). For A, B and C scale bars 50 *µ*m and 20 *µ*m for the close-up views (scale shown in 1 image applies to all images in the panel). **D)** Starch accumulation in Col Wt, *pds3*, *clb5,* and *spc1-2* roots analyzed by Lugol staining in 5- (5 d SLC) and 10-d-old (10 d SLC) seedling roots. Scale bar for A to D 0.5 mm (scale shown in 1 image applies to all images in the panel). **E)** Boxplot of the gravitropic response of *clb5* and *spc1-2* compared to *pds3* and Col Wt. Inner angles were measured 48 h after 90° rotation. The diagram illustrates the gravitropic response in Wt and *clb5* or *spc1-2* mutants. The box plots show the 25th and 75th percentiles of each group’s distribution, with the solid line indicating the median and T-shaped whiskers representing the minimum and maximum values. Asterisks indicate significant differences (*P* < 0.01) compared with their respective Wt, analyzed by 1-way ANOVA and Tukey’s HSD test. n.s. indicates no statistical significance (*P* ≥ 0.01). Total number of pooled individuals from 2 independent analyses is as follows: 25 for Wt, 84 for *pds3*, 42 for *clb5*, and 56 for *spc1-2*.

To investigate the effect of ACS1 on RAM organization, 5- and 10-d-old *clb5* and *spc1-2* seedlings grown under SLC were compared to Wt and *pds3*. While no major differences were observed in the RAM organization of 5-d-old *clb5* and *spc1-2* mutants compared to *pds3* and Wt plants (Supplementary Fig. S11A), 10-d-old mutants displayed a series of abnormalities in the stem cell niche and the CC, relative to *pds3* and Ler and Col Wt (Fig. 7A). Although RAM cell type identities appeared to be normal and no missing cell layers were detected, the QC cell alignment was frequently disturbed in *clb5* and *spc1-2,* resulting in disorganization of QC cells and irregular divisions in the columella initials (Supplementary Fig. S7Aix and x).

To further characterize these abnormalities, we analyzed the expression of the *WOX5* and *SCARECROW (SCR)* genes which regulate RAM establishment and maintenance (Di Laurenzio et al. 1996; Haecker et al. 2004). In Wt roots, *WOX5* is expressed in the QC, while *SCR* is expressed in the QC and endodermal cell lineage (Fig. 7, B and C). Confocal imaging of *proWOX5⸬GFP* and *proSCR⸬SCR:YFP* markers in 5-d-old *clb5*, *spc1-2*, and *pds3* mutants showed no differences compared to *pds3* and Wt (Supplementary Fig. S11, B and C). In 10-d-old *clb5* and *spc1-2* mutants, *SCR* and *WOX5* expression was clearly detected in QC cells (Fig. 7, B and C). However, we observed abnormal QC organization characterized by misaligned cells, where *WOX5* activity was also detected (Fig. 7Bvii and viii), in contrast to *pds3* and Wt (Fig. 7Bv and vi). This likely results from additional QC cell divisions. This indicates that while the stem cell identity appears preserved in the *clb5* and *spc1-2* QCs, ACS1 accumulation disrupts spatial organization in the stem cell niche resulting in irregular cell divisions, particularly affecting the columella initials. This does not affect provascular or lateral-root-cap/epidermis initials.

Given the observed columella defects in the *clb5* and *spc1-2,* starch accumulation was evaluated using Lugol staining, as functional CCs require amyloplasts that accumulate starch to facilitate proper gravitropic responses (Zhang et al. 2019). Both *clb5* and *spc1-2* mutants showed a reduced starch content in CC layers at 5 and 10 d compared to *pds3* and Wt (Fig. 7D). This suggests that amyloplast differentiation and starch accumulation are impaired in *clb5* and *spc1-2*. To assess the physiological impact of these changes, we analyzed gravitropic responses in 10-d-old *clb5*, *spc1-2*, *pds3*, and Wt seedlings after a 90° rotation. Both *clb5* and *spc1-2* displayed larger inner rotation angles (140° and 130°, respectively; *P* = 2.22e-16) in comparison to Wt (90°) and *pds3* (95°; *P* = 0.0099), indicating delayed gravitropic responses (Fig. 7E). Overall, these results demonstrate that ACS1 accumulation in *clb5* and *spc1-2* disrupts stem cell niche organization, alters CC morphology, impairs statolith formation, and delays gravitropic responses.

### Differential expression of the ACS1 precursor enzymes

To provide insight into the regulation of ACS1 accumulation within the SAM and RAM and further support ACS1's physiological relevance, the expression patterns of key enzymes required for ACS1 precursors synthesis were analyzed (i.e. deoxy-D-xylulose 5-phosphate synthase (DXS1), PDS3, and ZDS). DXS is part of the MEP pathway for carotenoid precursors synthesis (Cordoba et al. 2009), while PDS3 and ZDS are directly involved in ACS1 precursors synthesis (Supplementary Fig. S12A) (Alagoz et al. 2018).

**Figure 8.**
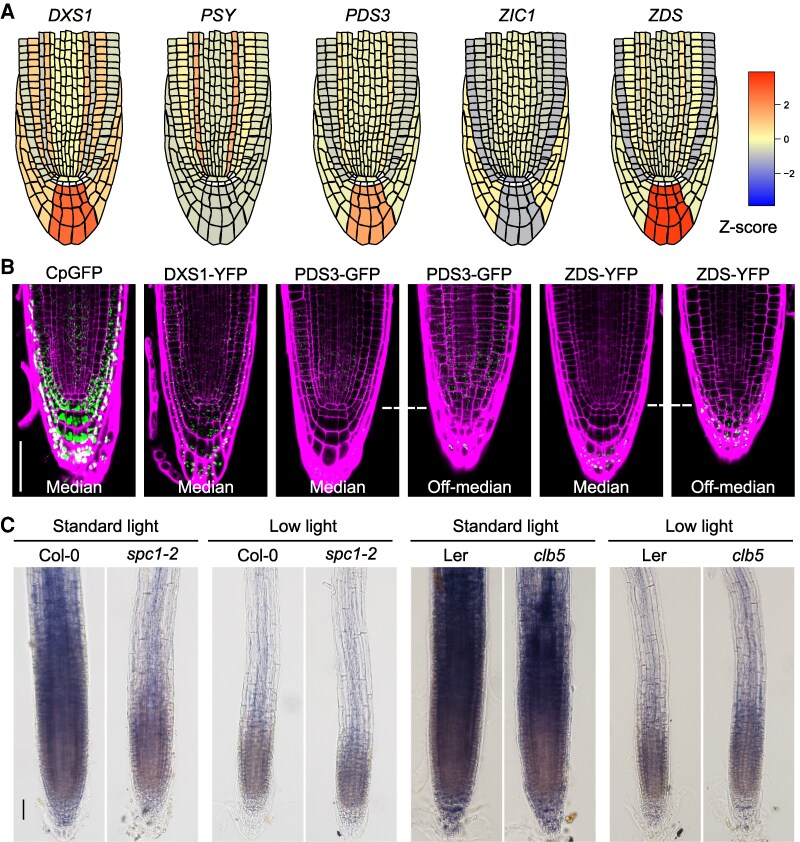
RAM expression of carotenogenic genes and proteins involved in ACS1 precursors. **A)** Diagrammatic representation of expression levels of the carotenogenic genes *DXS*, deoxyxylulose 5-phosphate synthase; *PSY*, phytoene synthase; *PDS3*, phytoene desaturase; *ZIC1*, zeta carotene isomerase and *ZDS*, ζ-carotene desaturase in the RAM. The color code corresponds to the expression levels according to the *Z*-score shown in the heat map. **B)** Localization patterns of the proteins from plastid marker *pro35S⸬RBCSctp:GFP* (CpGFP), *proDXS⸬DXS1:YFP* (DXS1*⸬*YFP), *proPDS3⸬PDS3-GFP* (*PDS3⸬GFP*), and *proZDS*:ZDS-YFP (ZDS*⸬*YFP) fusion proteins in the RAM of 10-d-old Wt seedlings. Images correspond to median (left) and off-median (right) transverse sections of the RAM. Cell membranes were stained with propidium iodine. Images are representative of 3 independent biological replicates *n* ≥ 5. Scale bar 50 *µ*m (scale shown in 1 image applies to all images in the panel). **C)** Distribution of singlet oxygen detected by nitroblue tetrazolium (NBT) staining in root tips of 10-d-old Col-0 and L*er* Wt, *clb5*, and *spc1-2* mutants. Scale bar for B and C, 0.5 mm (scale shown in 1 image applies to all images in the panel).

Single-cell transcriptomics data from the SAM showed that *DXS1*, *PSY*, *PDS3, ZIC1* (CRTISO-encoding gene), and *ZDS* expression levels are generally low but vary within the SAM (Tian et al. 2019; Sierra et al. 2022). Specifically, *PDS3* expression was lower in the SAM central zone and higher in the rib zone, whereas *ZDS* showed the opposite pattern, suggesting possible ACS1 accumulation regions (Supplementary Fig. S12B). Similar differential expression was observed in the RAM using publicly available high-resolution single-cell RNA-seq data from the Arabidopsis roots (Denyer et al. 2019), with higher levels of *DXS1* and *ZDS*, lower levels of *PDS3* and *PSY* and very low *ZIC1* expression in the CC (Fig. 8A).

To confirm the differential accumulation at the protein level, we generated transgenic lines expressing DXS1, PDS3, and ZDS under their native promoters, fused to YFP or GFP reporters. This resulted in the *proDXS1⸬DXS1:YFP, proPDS3⸬PDS3:GFP*, and *proZDS⸬ZDS:YFP* lines (Supplementary Fig. S13). We also included the *pro35S⸬RBCSctp:GFP* (*C-cpGFP*) control line for plastid localization (Wu et al. 2019). Due to difficulties in SAM visualization (Supplementary Fig. S12C), we focused on the RAM expression.

Consistent with our transcriptomic data, we detected low but detectable levels of these carotenogenic enzymes in the RAM. DXS1-YFP accumulates in plastids across most RAM tissues, including QC cells where the C-cpGFP marker protein was not detected (Fig. 8B), indicating its essential role throughout the RAM. In contrast, PDS3-GFP and ZDS-YFP proteins exhibited distinct expression patterns. ZDS:YFP was more abundant in the CC and lateral root cap, but nearly undetectable in the central cylinder, endodermis and cortex (Fig. 8B). Conversely, PDS3-GFP was present in the central cylinder, endodermis, and cortex but at lower levels in the columella (Fig. 8B). These patterns support the hypothesis that different *cis*-carotenoid intermediates accumulate in spatially distinct regions of the RAM and serve as precursors for the synthesis of different apocarotenoids.

Since our data suggest that ACS1 synthesis relies on both enzymatic and nonenzymatic cleavage of the ZDS substrates (phytofluene and ζ-carotenes) mediated by CCD4 and singlet oxygen species, we examined the spatial distribution of superoxide using nitroblue tetrazolium (NBT) staining. As has previously reported (Dunand et al. 2007), superoxide accumulation is evident in the meristematic zone of Wt root tips, with highest levels observed in the differentiation and elongation zones (Fig. 8C). Notably, this region coincides with a zone of higher PDS3 and lower ZDS expression (Fig. 8B), where ζ-carotene isomers may accumulate and potentially undergo oxidative cleavage, contributing to ACS1 synthesis. Consistently lower NBT accumulation is observed in CC where higher ZDS activity was detected and where low *ZIC1* transcript accumulates. Accordingly, with this model singlet oxygen species significantly decrease under low light conditions in Wt, *clb5* and *spc1-2* mutants, correlating with a reduction in the ACS1 synthesis that could explain the phenotypic reversion observed under these conditions.

## Discussion

Recent studies have revealed the diversity of carotenoid-derived compounds that act as hormones, signals, and growth regulators, including strigolactones, ABA, β-cyclocitral, and retinal (Moreno et al. 2021; Sierra et al. 2022). However, many apocarotenoids, particularly those derived from *cis*-carotenes, remain poorly understood. Functional analyses are limited by their low abundance and pleiotropic effects of carotenoid precursor disruptions, often leading to seedling lethality (Cazzonelli et al. 2020).

This study expands previous findings on the *cis*-carotene-derived apocarotenoid ACS1, showing that its over-accumulation disrupts early plastid biogenesis, causes severe developmental defects, and alters the expression of hundreds of nuclear genes related to plastid function and development providing a powerful tool to dissect its role more effectively. In *clb5* or *spc1-2* mutants, ACS1-associated leaf defects are partially or fully reversed either by CCD4 loss or low light, coinciding with the ACS1 precursors accumulation and supporting reduced ACS1 levels (Avendaño-Vazquez et al. 2014; Escobar-Tovar et al. 2021; McQuinn et al. 2023). Interestingly, morphological and transcriptional defects associated with ACS1 overaccumulation become more pronounced later in development, suggesting that while ACS1 presence may be required at specific stages, its sustained high levels are detrimental across multiple tissues. Notably, many of these defects are reversed when ACS1 levels decline, for example under low-light conditions. We manipulate ACS1 signal levels using low light to demonstrate its regulatory function in controlling plastid biogenesis, leaf, and root development. Our findings reaffirm that ACS1 is not a toxic bioproduct, but rather a physiologically relevant molecule. The low-light-induced recovery persists days after germination, indicating that ACS1 accumulation does not cause irreversible damage. These findings support the role of ACS1 as a signaling molecule and highlight the plasticity of leaf and root development. Moreover, in tomato, reduced ζ-carotene precursor levels disrupt floral meristem termination, reinforcing the physiological relevance of this signal under non-overexpressing conditions during floral development, which appears to be restricted to the meristem and possibly reliant on a tightly controlled gradient between specific cell types (McQuinn et al. 2023).

A major question that remains open is the identity of the primary targets of the ACS1 signal. The gene expression profiling at stage 1.02 of the *clb5* mutant showed many top DEGs relate to plastid functions, including PhANGs and housekeeping genes. In *clb5,* genes encoding components of PEP, NEP, and chlororibosomal proteins are expressed at much lower levels than in *pds3*, although some housekeeping genes like *POL1B* and *TOC132* maintain similar expression. Accordingly, plastid genome copy numbers in *clb5* and *spc1-2* are comparable to *pds3* and Wt plants, indicating that plastid DNA replication occurs similar to *pds3* and Wt seedlings. However, other early biogenesis functions such as plastid transcription, translation, and import of photosynthetic peptides are impaired. These data suggest that ACS1 accumulation arrests plastid biogenesis close to proplastid stage after plastome proliferation, earlier than several reported maize and *Arabidopsis* albino mutants, or NFZ-treated seedlings (Loudya et al. 2020; Kendrick et al. 2022).

Unlike other albino mutants or chemically impaired seedlings, *clb5* mutants with ACS1 accumulation do not show the typical compensatory upregulation of nuclear-encoded plastid housekeeping genes (Hajdukiewicz et al. 1997; Loudya et al. 2020). Instead, the *clb5* expression profile resembles that of the *cue8 gun1* double mutant, which arrests near proplastid stage (Loudya et al. 2020). However, the plastids in the ACS1-accumulating seedlings sustain DNA replication without causing lethality, as developmental resumes once ACS1 levels decrease under LL. Together, these findings suggest that *clb5* undergoes developmental arrest at a very early stage of plastid differentiation.

Nevertheless, the primary target(s) of ACS1 remain unknown, and it is unclear whether ACS1 directly triggers changes in plastid-related gene expression that lead to organelle arrest and, consequently, shoot, and root defects. An alternative possibility is that ACS1 acts on targets involved in cell differentiation or organelle development, which subsequently affect plastid biogenesis. This is particularly relevant given that *clb5* and *spc1-1* mutants exhibited numerous DEGs unrelated to plastid functions, including genes involved in hormone signaling and key transcription factors critical for leaf and flower development, some of which may be primary ACS1 targets.

Previous studies showed that the leaf morphological defects in *clb5* and *spc1-2* are phenotypically indistinguishable from those observed in Wt plants treated with plastid translation inhibitors or in certain ribosomal protein mutants, all known to trigger biogenic retrograde signaling (Tiller and Bock 2014; Escobar-Tovar et al. 2021). Plastid translation is significantly reduced in *clb5* and *spc1-2* due to the downregulation of over half of the plastid ribosomal protein genes. Given the epistatic nature of the plastid defects over leaf development, it has been concluded that at least some of the morphological defects associated with ACS1 accumulation, such as altered leaf architecture, result from impaired expression of plastid biogenesis-related genes (Escobar-Tovar et al. 2021). Other plastid differentiation mutants that activate plastid retrograde signaling also showed anatomical abnormalities, including altered leaf and flower morphogenesis across diverse species (Aluru et al. 2009; Tiller and Bock 2014). In contrast, many developmental mutants with altered leaf morphology, such as the *pinoid* or *kanadi,* do not display major defects in plastid biogenesis (Kerstetter et al. 2001; Mudgett et al. 2023). However, these findings relate only to leaf-specific phenotypes during ACS1 overaccumulation and not ACS1’s broader physiological roles (e.g. meristem maintenance, flower development or roots). Determining whether plastid-related genes are direct ACS1 targets will require further investigation. A major limitation is the pleiotropic effects resulting from the inhibition carotenoid biosynthesis in mutants (e.g. *pds3*) or through chemical inhibitors (NFZ), which may mask the developmental consequence of ACS1 absence. Alternative approaches, such as inducing transient ACS1 accumulation using different light conditions and analyzing gene expression at specific developmental stages, will be needed.

Recent research underscores the central role of plastid retrograde signaling in coordinating plastid biogenesis and function, with cellular and developmental programs. During normal plastid biogenesis, sequential production of positive or negative retrograde signals communicates organelle differentiation status at specific stages, coupling plastid maturation with tissue differentiation and organogenesis (Andriankaja et al. 2012; Loudya et al. 2020, 2021; Kendrick et al. 2022). Disruption of plastid differentiation can sustain a specific retrograde signal, associated with a specific gene expression profile, while preventing the synthesis of subsequent signals, thereby arresting tissue development.

In this context, the gene expression profile and morphological defects in mutants with elevated ACS1 levels align with this model, particularly during stages of leaf expansion. The early plastid development arrest observed in *clb5* likely underlies the pleiotropic defects not only in shoots but also in roots, distinguishing *zds* from several other studied albino mutants. However, whether ACS1 itself acts as a signal translocating from plastids to the nucleus to modulate gene expression remains an open question for future research.

SAM and RAM serve as reservoirs of cells for organ formation, and their proper regulation is essential to maintain the balance between cell renewal and differentiation. This regulation is tightly controlled by a network of transcription factors and phytohormones. Our analysis demonstrated that in the *clb5* mutant, the WUS-CLV3 signaling system is disrupted, indicating compromised SAM homeostasis due to the sustained accumulation of ACS1, which hinders the development of new lateral organs. Specifically, *CLV3* transcription levels significantly decrease during *clb5* seedling development compared to Wt or Wt seedlings treated with NFZ. WUS and CLV3 are highly conserved regulators that are part of the CLV-WUS network, required for maintaining SAM homeostasis during post-embryonic development (Brand et al. 2000). The WUS-CLV3 signaling system regulates SAM activity by balancing auxin and cytokinin gradients, partially by modulation of expression of auxin and cytokinin signaling genes (Pernisová and Vernoux 2021). This balance is critical for the proper patterning and the emergence of the leaf primordia in the SAM's peripheral zone, where auxin accumulation promotes primordium formation (Galvan-Ampudia et al. 2020). In *clb5,* a progressive loss of *PIN1:GFP* and *proDR5⸬GUS* expression was observed, correlating with disrupted auxin gradients at the SAM. This disruption aligns with the downregulation of several auxin transport and signaling genes in *clb5*.

Auxin also modulates SAM homeostasis by downregulating *CLV3* expression through the AUXIN RESPONSE FACTOR 5**/**MONOPTEROS **(**ARF5**/**MP) TF (Luo et al. 2018). Interestingly, the transcriptomic analysis showed that *ARF5* accumulates at higher levels in *clb5* compared to *pds3*, particularly at later developmental stages. This finding is intriguing and warrants future investigation.

Additionally, this study demonstrates that ACS1 accumulation alters the development of non-photosynthetic tissues, a phenomenon not previously reported in other carotenoid-deficient mutants, such as *pds3*. These defects include shorter RAM lengths, abnormal columella development, reduced starch accumulation in CC, impaired normal gravitropic responses and decrease LR initiation. Notably, previous research has shown that NEP-mediated transcription and translation are critical for primary and LR growth and for maintaining stem cell patterning in LR (Hricová et al. 2006; Nakata et al. 2018). Our findings further support the essential role of plastid status in root development.

Gravity sensing is important for plant nutrient absorption and environmental responses, initiated with the sedimentation of the amyloplasts within the CC (Morita 2010). Reduced starch accumulation in amyloplasts has been linked to altered root gravitropism, highlighting the direct role of starch content in regulating this response (Sack 1997). CCs differentiate from the columella stem cell initials, a process tightly coordinated with the biogenesis of amyloplasts from proplastids. During differentiation, statoliths (specialized amyloplasts) in CCs accumulate starch and develop specific morphology and functionality. However, the molecular mechanisms governing this differentiation process remain poorly understood (Morita 2010; Chen et al. 2023). In *clb5* and *spc1-2* mutant roots, the number of starch granules in CC appeared reduced, suggesting disrupted amyloplast differentiation. Various apocarotenoid signals and hormones influence root development. For example, ABA, strigolactones, and retinal influence root architecture, while β-cyclocitral promotes cell division in the root meristem and enhances root branching (Dickinson et al. 2019, 2021; Moreno et al. 2021). Interestingly, our findings suggest that ACS1 plays a distinct role in columella development that does not overlap with these other apocarotenoids. This is evident because the *pds3* mutant, which disrupts β-carotene-derived apocarotenoid synthesis, does not exhibit the altered CC morphology or gravitropic defects observed in *clb5*. Therefore, the mechanism by which ACS1 impacts the organization of the stem cell niche and columella initials remains unclear and requires further investigation.

To support optimal plant growth, continuous developmental adjustments are necessary in response to environmental and metabolic cues. By modulating the accumulation of ACS1 levels, our study further supports the potential link between plastid status and the regulation of broad aspects in plant development. This regulation impacts not only photosynthetic tissues but also other organs such as SAM and RAM maintenance. Taken together, our findings further support the notion that ACS1 synthesis may occur in a tissue- or organ-specific manner, not only in flowers as previously suggested (McQuinn et al. 2023), but also in other organs such as roots. High levels of this carotenoid-derived signal, would maintain an early differentiation plastid status and delay further development in leaves and CC. In this context, we propose that ACS1 likely plays an important role in maintaining meristem integrity and proper organ differentiation. This is consistent with the developmental alterations observed in tomato floral meristems when the ACS1 precursors were artificially decreased (McQuinn et al. 2023), and suggests broader implications for plant growth and adaptive response mechanisms.

## Materials and methods

### Plant material and growing conditions

This work used *Arabidopsis thaliana* ecotypes Columbia-0 (Col-0) and Lansberg erecta (L*er*), along with previously reported mutants: *clb5* in Ler background (Gutiérrez-Nava et al. 2004), *pds3-1* (Qin et al. 2007), and *spc1-2*/SALK_033039 (Dong et al. 2007) in Col-0. Reporter lines included *pWOX5⸬GFP*, *proSCR⸬SCR:GFP*, *proDR5⸬GUS*, and *proWUS⸬GUS* (Heidstra et al. 2004; Bäurle and Laux 2005; Sarkar et al. 2007; Scarpella et al. 2010). The *proCLV3⸬CLV3:GUS* and *Ccp:GFP* constructs were kindly provided by Dr S. de Folter and Dr R. Bock, respectively (Wu et al. 2018). Seedlings were grown on 1× GM media (Murashige and Skoog with Gamborg vitamins, Phytotechology Labs, USA) with 1% (w/v) sucrose and 0.8% (w/v) phytoagar, stratified at 4 °C for 4 d. and growth under 16:8 h light:dark cycle at 22 °C, either at 100 *µ*mol m^−2^ s^−1^ (standard light conditions, SLC) or 5 *µ*mol m^−2^ s^−1^ (low light LL). For LL treatments, seedlings were transferred from SLC to LL at the indicated times points and evaluated on d 21. For dark treatments, 15-d-old seedlings grown under SLC were transferred to darkness for 15 d and assessed at 30 d. Mature plants were grown in peat moss:perlite:vermiculite (5:3:2) mix (Sunshine mix 3, Sun Gro Horticulture, Agawam, USA; Sun Gro Horticulture; Agrolita, Tlalnepantla, Mexico) supplemented with Osmocote (1.7 kg/m^3^, Everris, Geldermalsen, Netherlands) under 16:8 h photoperiod at 22 °C and 65 *µ*mol m^−2^ s^−1^.

Reporter lines were crossed with heterozygous *clb5, spc1-2*, and *pds3* backgrounds. Transgene presence and mutations segregation were confirmed via albino phenotype and GUS staining. The *clb5* DR5:GUS reporter line was previously reported (Avendaño-Vazquez et al. 2014).

### Hormone and norflurazon treatments

For hormone treatments, heterozygous seeds of *pds3*, *clb5*, *spc1-2*, and homozygous Col-0 and Ler were germinated on GM medium and transferred 6 d after germination to either control GM or GM supplemented with 0.1 *µ*m or 0.2 *µ*m IAA, 0.4 *µ*m GR24 (Gomez-Roldan et al. 2008), or 0.1 *µ*m or 0.5 *µ*m ABA (Sigma-Aldrich, St. Louis, USA). Seedlings were grown under SLC, and root phenotypes were analyzed at 15 d post-germination. For dark hormone treatments, seedlings were grown under SLC for 15 d, then transferred to GM containing 0.1 *µ*m IAA and incubated in darkness for 15 d. Morphology was evaluated at 30 d. For NFZ treatments, seedlings were grown on GM supplemented with 0.5 *µ*M NFZ (Sigma-Aldrich, St. Louis, USA) at the indicated time points.

### Histochemical GUS staining and sample clearing

Seedlings were fixed in 90% acetone for 2 h at −20 °C, then incubated in GUS staining solution (100 mm NaPO_4_ pH 7.2, 0.1% Triton X-100, 10 mm EDTA, 5 mm potassium ferrocyanide, 5 mm potassium ferricyanide for at least 20 min. Samples were vacuum infiltrated with GUS solution containing 1 mg/mL X-GLUC (Jefferson 1987), and incubated at 37 °C for 5 h (*proCLV3⸬CLV3:GUS*, *pDR5⸬GUS*) or overnight (*proWUS⸬GUS*, *pDR5⸬GUS*). Pigments were removed with 70% ethanol. For clearing and mounting, tissues and roots were cleared in 0.24 N HCl and 20% methanol at 65 °C for 1 h, neutralized with 7% NaOH in 60% methanol for 20 min, washed in 70% ethanol, through graded ethanol dilutions (40%, 20%, 10%) and mounted in 50% glycerol with 2% DMSO.

### Lugol and NBT staining

Five- and 10-d-old seedlings were fixed in paraformaldehyde 4% in PBS1X, (pH 7.2), cleared 5 d using ClearSee (10% xylitol, 15% sodium deoxycholate and 25% urea), then stained with Lugol's solution (Sigma-Aldrich, St. Louis, MO, USA) for 2 min, and rinsed with distilled water. Samples were mounted and observed under an optical microscope. For NBT staining, 10-d-old seedlings were vacuum infiltrated in a 20 mm phosphate buffer (pH 7.2) containing 0.5 mg/mL^−1^ nitroblue tetrazolium (NBT, Sigma Aldrich) for 10 min. The reaction was stopped with 10 mm phosphate buffer. Pigments were removed using 70% ethanol followed by rehydration through graded ethanol series and mounted in 50% glycerol + 2% DMSO and imaged under optical microscope.

### Morphometric analyses and gravitropic response quantification

The LRP density was obtained counting the LRP in the lateral root formation zone (Dubrovsky and Forde 2012), normalized per millimeter of parent root. *l_LRI_*, was calculated as the number of LRs and LRP per parent root segment corresponding to 100 cortical cells in a cell file (Dubrovsky et al. 2009). For gravitropism, seedlings were grown vertically on GM media under SLC for 10 d, and the position of the root tip was recorded. After rotating the plants 90 degrees, root tip position was recorded again 48 h later. The internal angle was measured using ImageJ (Schneider et al. 2012).

### Light and confocal microscopy

Bright field images were acquired with a Nikon SMZ1500 stereomicroscope. For Confocal microscopy roots and aerial tissues were incubated in 0.1% propidium iodide (PI, Sigma-Aldrich, Saint Louis, MO., USA) for 10 min. Images were acquired with an Olympus *Fluoview* FV1000 multi-photon confocal microscope with a 30× objective and a Kalman filter (*n* = 4). Excitation/emission: GFP and YFP: 488 nm/505 to 525 nm; PI: 543 nm/560 to 660 nm; chlorophyll: 635 nm/BA655 to 755 nm. Maximum intensity projections were generated in FIJI (ImageJ Bethesda, Maryland, USA).

### Molecular cloning

To generate the *proDXS1⸬DXS:YFP* construct, a 4,000 bp genomic fragment, comprising 1,800 bp of the upstream regulatory region and the *DXS1* coding sequence including introns, was amplified using CLAp1800GW and CLANSTP primers (Supplementary Table S5). For the *proPDS3⸬PDS3:GFP* fusion, a 5,476 bp fragment (1,800 bp promoter and the full *PDS3* coding region with introns) was amplified using PDS3BPFw and PDS3nstpRev primers (Supplementary Table S5). The *proZDS⸬ZDS:YFP* construct was obtained by amplifying a 5,592 bp fragment encompassing the 2,243 bp intergenic region, 5′ UTR, and *ZDS* coding sequence with introns using ZDSFw and ZDSRv primers (Supplementary Table S5). PCR fragments were cloned into Gateway-compatible binary vectors, pMDC204 (for *PDS3*) or pHGY (for *DXS1* and *ZDS*), and introduced into Col-0 plants via *Agrobacterium tumefaciens*-mediated floral dip transformation (Clough and Bent 1998). At least 3 homozygous transgenic lines per construct were selected and analyzed for expression.

### RNA extraction and quantitative real-time polymerase chain reaction

Total RNA was extracted using TRIzol (Thermo Fisher Scientific, Carlsbad CA, USA), following the manufacturer instructions. Three µg of RNA were reverse transcribed with M-MLV reverse transcriptase (Invitrogen) and oligo dT primers. Quantitative real-time PCR were performed using Luna Universal qPCR Master Mix (NEB, Ipswich, MA, USA) on a CFX96 system (Bio-Rad Laboratories). Primers for TOC132, POL1B and ACT7 are listed in Supplementary Table S5. Experiments were conducted in biological triplicates with technical duplicates.

### RNA seq analysis

RNA-seq was performed from 8-d-old *clb5* and *pds3* seedlings (cotyledon stage 1.0), using 3 independent biological replicates. Library preparation and sequencing followed Escobar-Tovar et al. (2021). Reads were mapped to annotated genes in the *Arabidopsis thaliana* TAIR10 genome using STAR V2.7.10a. Differentially expressed genes (DEGs) were identified via the IDEAMEX platform (Jiménez-Jacinto et al. 2019) using edgeR, DEseq2, Limma, and NOIseq, applying thresholds of log_2_ fold-change ≥1.5, FDR <0.05 and adjusted *P*-value <0.05. Plots were generated in R-Studio. Raw reads are available in the NCBI BioProject PRJNA63880 (https://www.ncbi.nlm.nih.gov/bioproject/?term=PRJNA638809).

### Determination of chloroplast DNA copy number

Genomic DNA was extracted from 15-d-old seedlings grown on GM medium using the CTAB method. Chloroplast DNA copy number was quantified by qPCR, using 2 nuclear genes (*HEMA1* and *RpoTp*) and 3 single copy plastid genes (*rbcL*, *rpoA*, and *ycf2*) as targets. Each qPCR was performed using 25 ng of genomic DNA for nuclear genes and a 100-fold dilution for plastid genes. Reactions were done with Luna Universal qPCR Master Mix (NEB, Ipswich, MA, USA) on a CFX96 system (Bio-Rad). Relative plastid DNA abundance was calculated via the 2−ΔΔCT method, normalized to the nuclear genes. Primer sequences are listed in Supplementary Table S5. Experiments were conducted in triplicates with technical duplicates.

### Data analysis

Boxplots (25th and 75th percentiles, median, and whiskers for maximum and minimum values) and violin plots were used to visualize data distribution. Parametric 1-way ANOVA (*α* = 0.01) followed by Tukey HSD test, was used for multiple and pairwise comparisons (Supplementary Table S6). Normal distribution was assessed via the Shapiro-Wilk test (*α* = 0.05). Where indicated, the non-parametric Kruskal–Wallis test (right-tailed *χ*² *a* = 0.05) and post hoc Dunn's test (*a* = 0.005) and fixed-effects 2-way ANOVA with Type III sums of squares were applied.

### Accession numbers

The accession numbers of the major genes/proteins mentioned in the paper are shown in Supplementary Table S7.

Sequence data from this article can be found in the GenBank/EMBL data libraries under accession numbers PRJNA638809 (https://www.ncbi.nlm.nih.gov/bioproject/?term=PRJNA638809).

## Supplementary Material

kiaf414_Supplementary_Data

## Data Availability

Data set can be found in *GEO:* GSE152252.

## Supplementary data

The following materials are available in the online version of this article.

**Supplementary Figure S1.** Phenotypic recovery of leaf morphology in seedlings transferred to low light.

**Supplementary Figure S2.** Differentially expressed genes in the *clb5* mutant in 2 developmental stages.

**Supplementary Figure S3.** Expression pattern of the auxin response DR5 marker.

**Supplementary Figure S4.** Effect of auxins on the *clb5* and *spc1-2* leaf morphology.

**Supplementary Figure S5.** Leaf development of *clb5* and *spc1-2* mutants in response to auxins in the dark without sucrose.

**Supplementary Figure S6.** Primary root morphology in ACS1-accumulating seedlings.

**Supplementary Figure S7.** The RAM architecture defects in the *clb5* mutant revert under low light.

**Supplementary Figure S8.** Auxin response in the primary root in ACS1-accumulating seedlings.

**Supplementary Figure S9.** Response of the primary root to hormonal treatments.

**Supplementary Figure S10.** Effect of ABA and SL supplementation on lateral root development in ACS1-accumulating mutants.

**Supplementary Figure S11.** Analysis of RAM and QC structure and expression QC markers.

**Supplementary Figure S12.** Expression patterns in carotenogenic enzymes in the SAM.

**Supplementary Figure S13.** Expression pattern of p*DXS1⸬DXS1:YFP,* p*PDS3⸬PDS3:GFP* and p*ZDS⸬ZDS:YFP* lines.

**Supplementary Table S1.** DEGs shared between *clb5* and *pds3* at 1.0 (cotyledon) and 1.02 (leaf) stages in the *clb5* mutant compared to *pds3* mutant.

**Supplementary Table S2.** Gene expression data for heatmap in Fig. 3B, *Z*-score values.

**Supplementary Table S3.** Gene expression data for heatmap in Fig. 4A, *Z*-score values.

**Supplementary Table S4.** RAM length of wild-type and albino mutants grown under standard light and low light conditions.

**Supplementary Table S5.** Sequence of the oligonucleotides used in this study.

**Supplementary Table S6.** Fixed-Effects 2-way ANOVA for Root length for wild type and carotenoid deficient mutants.

**Supplementary Table S7.** Accession numbers of the major genes/proteins mentioned in the paper.

## Dive Curated Terms

The following phenotypic, genotypic, and functional terms are of significance to the work described in this paper:

PDS3 Gramene: AT4G14210
PDS3 Araport: AT4G14210

WOX5 Gramene: AT3G11260
WOX5 Araport: AT3G11260

CRTISO Gramene: AT1G06820
CRTISO Araport: AT1G06820

ACT7 Gramene: AT5G09810
ACT7 Araport: AT5G09810

PI Gramene: Propidium iodide
PI Araport: Propidium iodide

PIN1 Gramene: AT1G73590
PIN1 Araport: AT1G73590

CLV3 Gramene: AT2G27250
CLV3 Araport: AT2G27250

toc132 Gramene: AT2G16640
toc132 Araport: AT2G16640

CCD4 Gramene: AT4G19170
CCD4 Araport: AT4G19170

ZDS Gramene: AT3G04870
ZDS Araport: AT3G04870

SLC Gramene: standard light conditions
SLC Araport: standard light conditions

LRP Gramene: lateral root primordium
LRP Araport: lateral root primordium

DXS1 Gramene: AT4G15560
DXS1 Araport: AT4G15560

NEP Gramene: nuclear encoded RNA polymerase
NEP Araport: nuclear encoded RNA polymerase

NCBI Gramene: https://www.ncbi.nlm.nih.gov
NCBI Araport: https://www.ncbi.nlm.nih.gov

PSY Gramene: AT5G17230
PSY Araport: AT5G17230

ACS1 Gramene: apocarotenoid signal 1
ACS1 Araport: apocarotenoid signal 1

pinoid Gramene: mutant
pinoid Araport: mutant
