# Supplementary material for: Apocarotenoid signaling regulates meristem activity and shapes shoot and root lateral organ formation in Arabidopsis
Source: Plant Physiol. 2025 Sep 24;199(2):kiaf414. doi: 10.1093/plphys/kiaf414 (PMC12508801; doi:10.1093/plphys/kiaf414)
Supplement: kiaf414_Supplementary_Data [file kiaf414_supplementary_data.zip › Supplementary Data.pdf]

Supplementary Figures

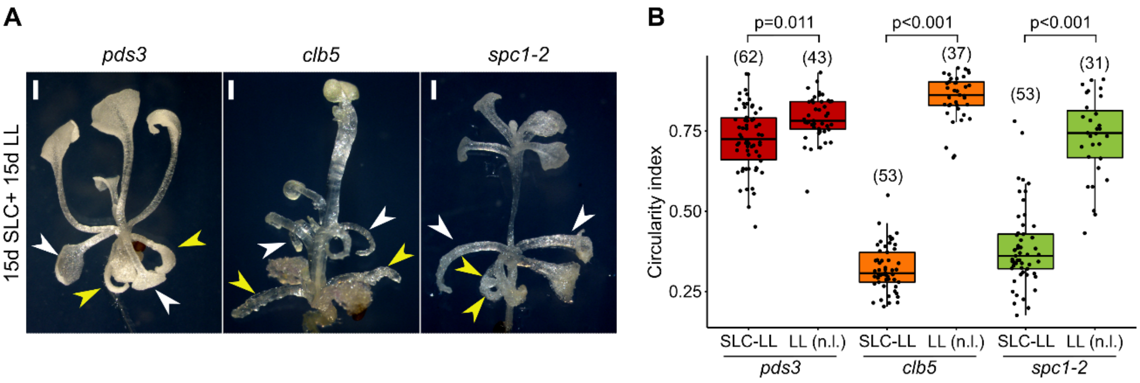

Supplementary Figure S1. Phenotypic recovery of leaf morphology in seedlings transferred to low light. (A) Phenotypes of 30-day-old plants grown under SLC for 15 days and transferred to LL for 15 days. Arrows indicate the first (1st and 2nd, yellow arrows) and second (3rd and 4th, white arrows) pair of leaves.

(B) Boxplot of the leaf circularity index 4π (leaf area/leaf perimeter^2) from *pds3*, *clb5* and *spc1-2* seedlings in SLC-LL and LL new leaves (n.l.) as shown in Figure 2B. Boxes represent the 25th and 75th percentiles, and the solid line indicates the median, and the whiskers denote the minimum and maximum values of each group. Statistical significance between genotypes was determined by one-way ANOVA followed by Tukey´s HSD test (p<0.01). Total number of pooled individuals is indicated in parenthesis and was determined from four independent analyses. Scale bar = 0.5 mm (scale shown in one image applies to all images in the panel).

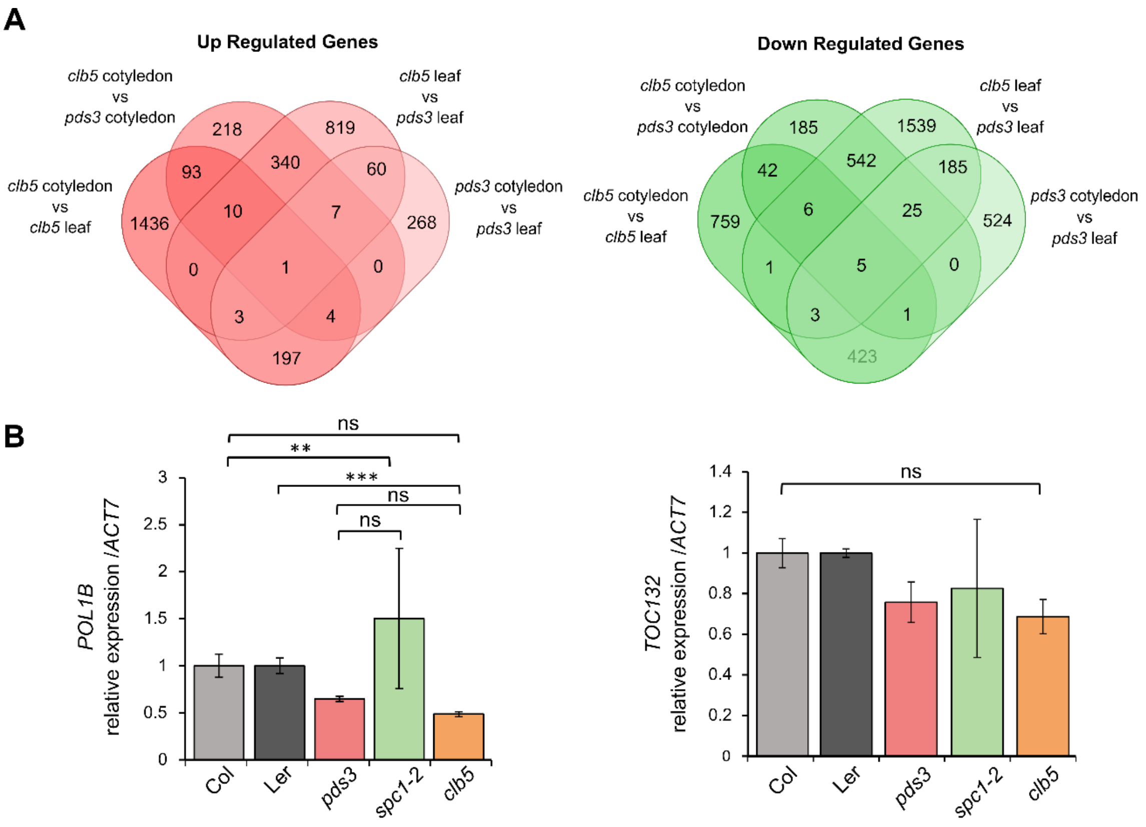

Supplementary Figure S2. Differentially expressed genes in the *clb5* mutant in two developmental stages. (A) Venn diagrams show the comparisons of the DEGs in *clb5* compared with *pds3* mutants at the two development stages 1.0 and 1.02 (the cotyledon stage or 1.0 was used as reference). (B) qRT-PCR analysis of *POL1B* and *TOC132* gene expression in Col-0 (Col) and Ler wild-type, *pds3*, *spc1* and *clb5* mutants. Expression levels are shown relative to *Actin 7* (*ACT7*). Bars represent means ± SD of triplicate biological replicas, each with two technical replicates. No significant differences were detected by one-way ANOVA (*, $P < 0.05$).

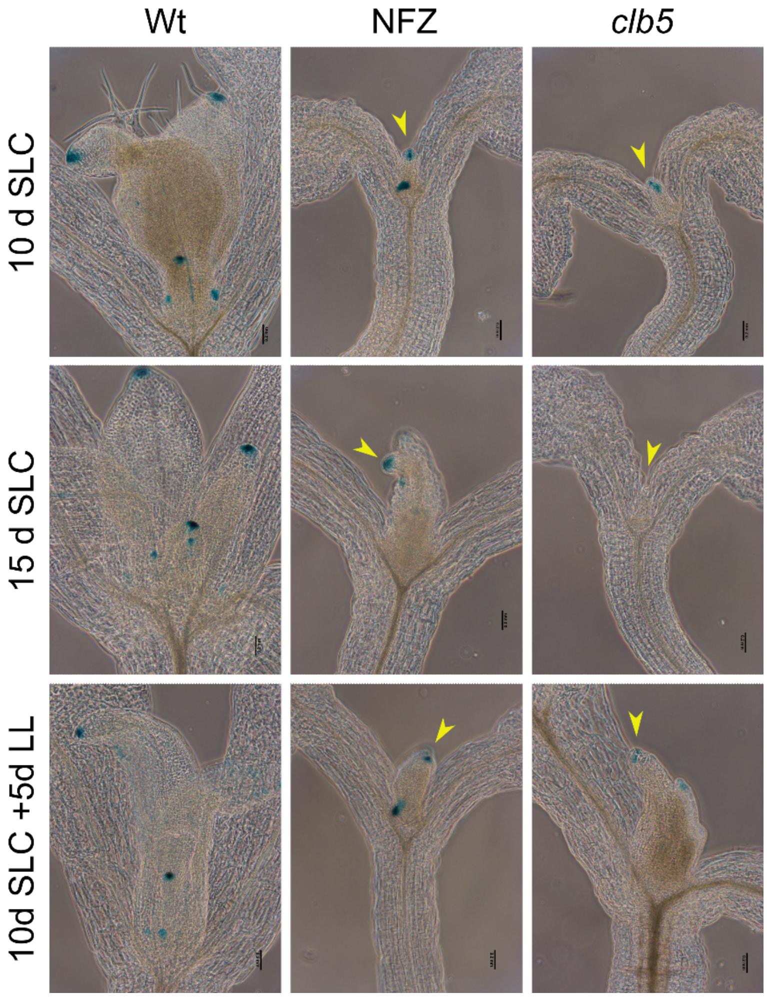

Supplementary Figure S3. **Expression pattern of the auxin response DR5 marker**. GUS expression pattern of the *proDR5::GUS* synthetic auxin marker from Ler (Wt), Ler treated with NFZ (NFZ) and *clb5* seedlings grown in standard light conditions (SLC, 100 µmol m^{-2} sec^{-1}) for 10 days, 15 days, or in SLC for 10 days and 5 day under low light (LL, 5 µmol m^{-2} sec^{-1}) (10+5). Yellow arrows indicate to the tip of the leaves. Scale bar corresponds to 0.2 mm.

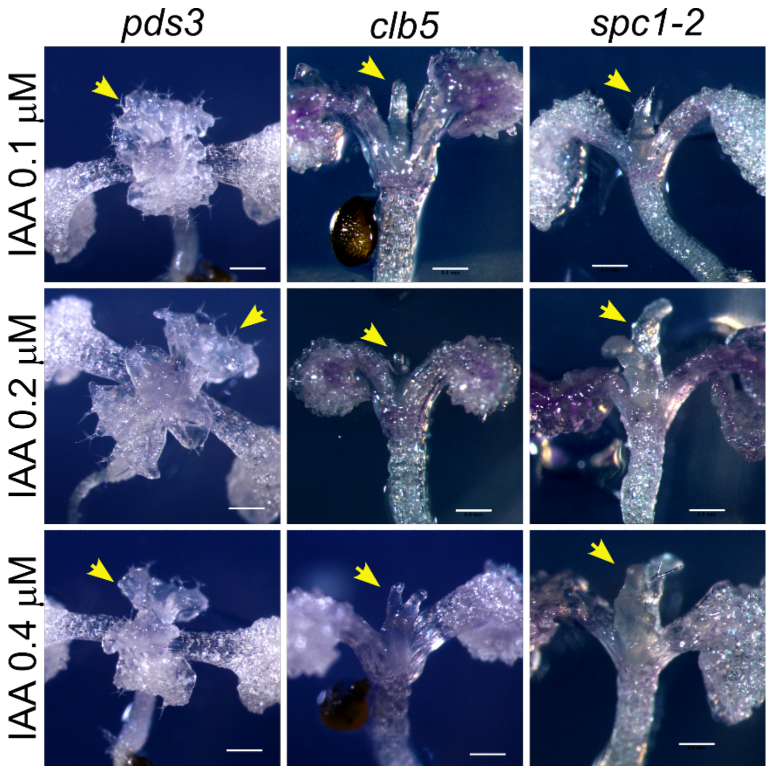

Supplementary Figure S4. **Effect of auxins on the *clb5* and *spc1-2* leaf morphology**. Leaf morphology of the 15-day-old *pds3*, *clb5* and *spc1-2* seedlings grown in GM medium or GM supplemented with 0.1 µM, 0.2 µM or 0.4 µM IAA. Seedlings were germinated in GM medium in standard light conditions (SLC 100 µmol m^{-2} sec^{-1}) and transferred to GM or GM+IAA after 6 days. Images show representative seedlings (n=15, three biological replicates). Scale bar corresponds to 0.5 mm.

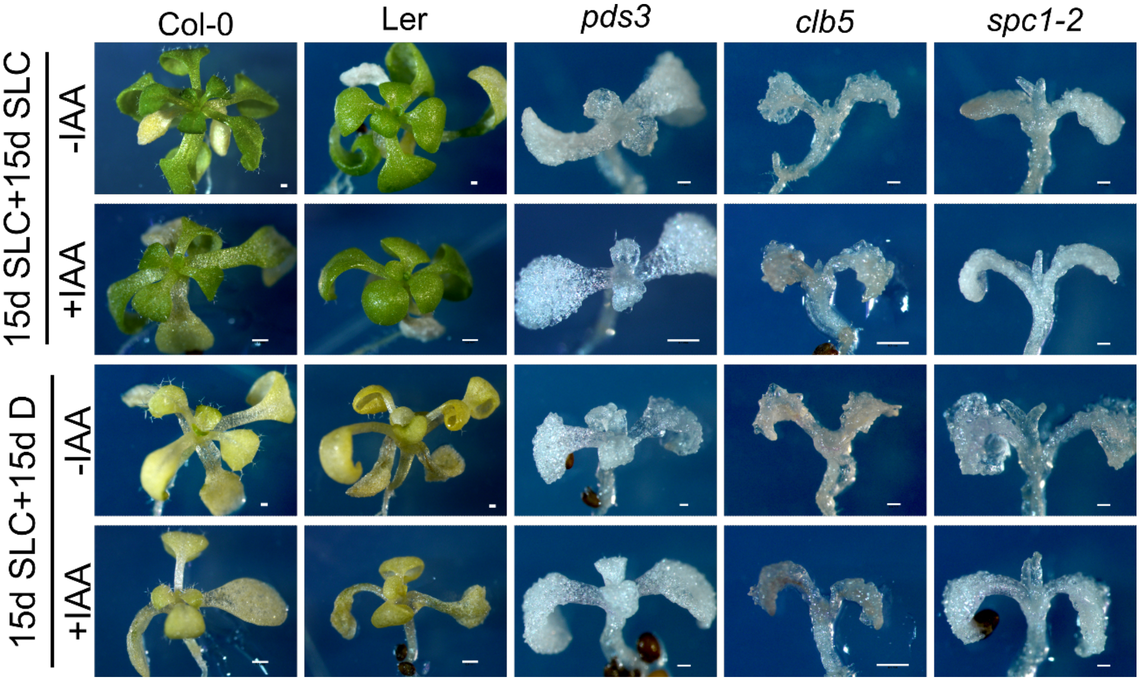

Supplementary Figure S5. Leaf development of *clb5* and *spc1-2* mutants in response to auxins in the dark without sucrose. Phenotypes of the 30-day-old Col-0, Ler, *pds3*, *clb5* and *spc1-2* seedlings initially grown for 15 days on media supplemented with 1% sucrose (+Suc) under standard light conditions (SLC) and then transferred to media without sucrose (-Suc) with (+) or without (-) auxin (IAA) and maintained in SLC or in the dark (D). Scale bar corresponds to 0.5 mm.

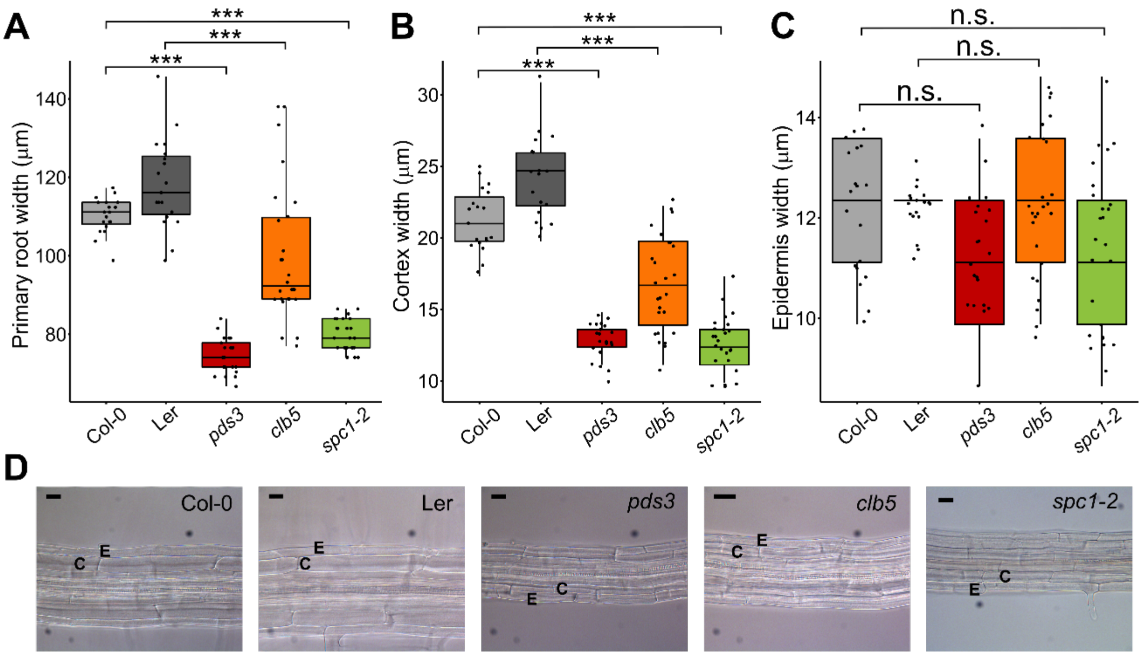

Supplementary Figure S6. Primary root morphology in ACS1-accumulating seedlings. Quantification of the primary root (A), cortex (B), and epidermis (C) cell widths in Col-0 and Ler Wt seedlings, and carotenoid-deficient *pds3*, *clb5* and *spc1-2* mutants. Cell width was measured in the differentiated zone of 12-day-old roots. Asterisks denote statistically significant differences compared to the respective Wt and letters indicate significant differences from pairwise comparisons. Boxes represent the 25th and 75th percentiles, and the solid line indicates the median, and the whiskers denote the minimum and maximum values of each group. Statistical analysis was performed by one-way ANOVA followed by Tukey´s HSD test (P <0.01); n.s. denotes no statistically significant differences (P≥0.01). Total number of pooled individuals from two independent analyses: 20 for Col-0, 18 for Ler, 20 for *pds3*, 26 for *clb5*, and 25 for *spc1-2*. (D) Representative images of the primary root differentiation zone from Col-0 and Ler and the carotenoid deficient *pds3*, *clb5* and *spc1-2* mutants. Epidermis (E) and cortex (C) cells are indicated. Scale bar corresponds to 20 mm.

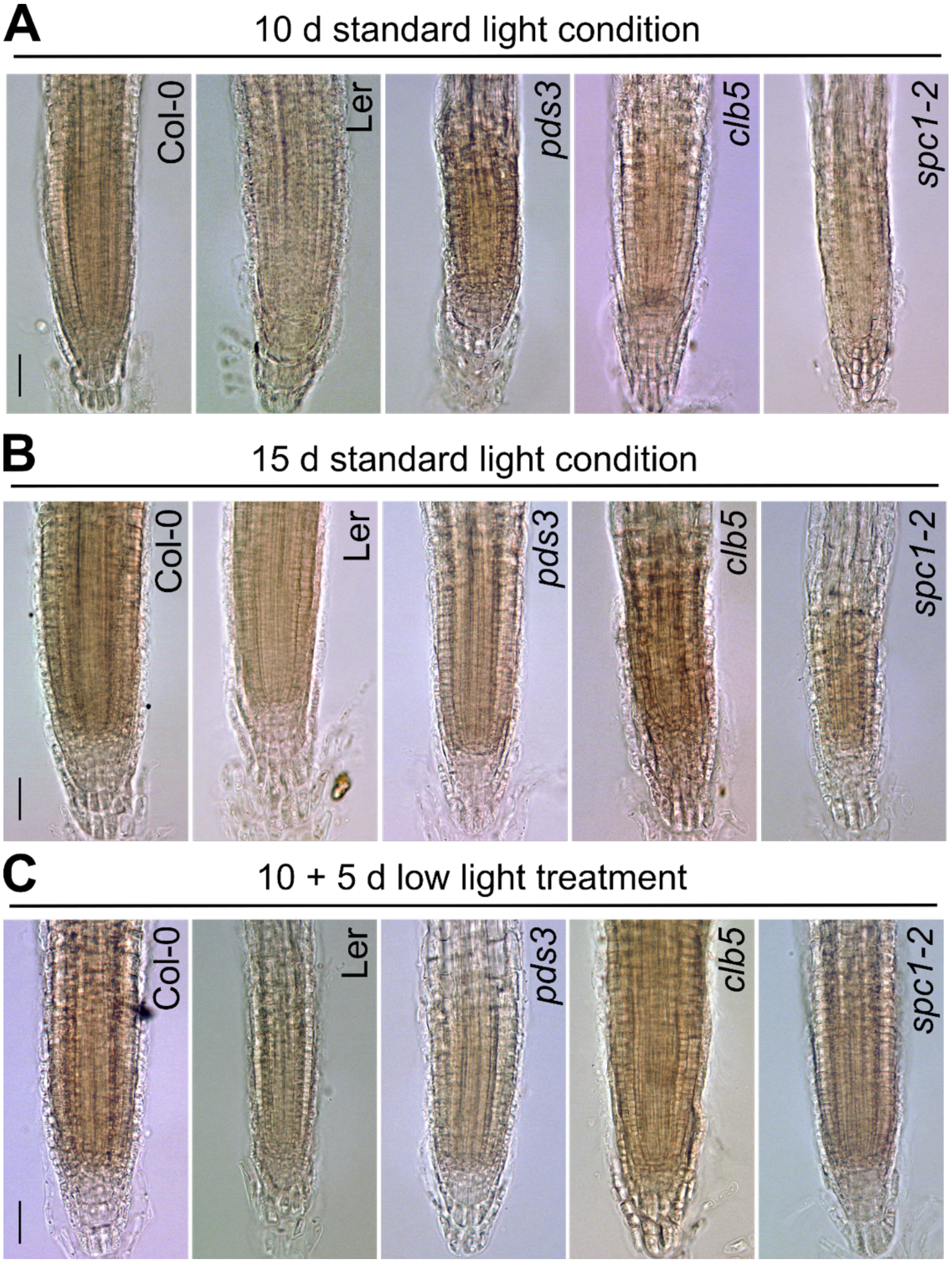

Supplementary Figure S7. The RAM architecture defects in the *clb5* mutant revert under low light. (A) Representative images of root tips from 10-day-old, (B) 15-day-old seedlings grown under standard light condition, and (C) 10-day-old seedlings grown in standard light condition and then transferred to low light for 5 days (10+5). Images are shown for Col-0, Ler, *pds3*, *clb5* and *spc1-2.* Morphological defects observed in the RAMs of *clb5* and *spc1*-2 at 10 (A) and 15 (B) days were reversed after 5 days of growth under low light conditions (C). Scale bar for A-C corresponds to 50 mm (scale shown in one image applies to all images in the panel).

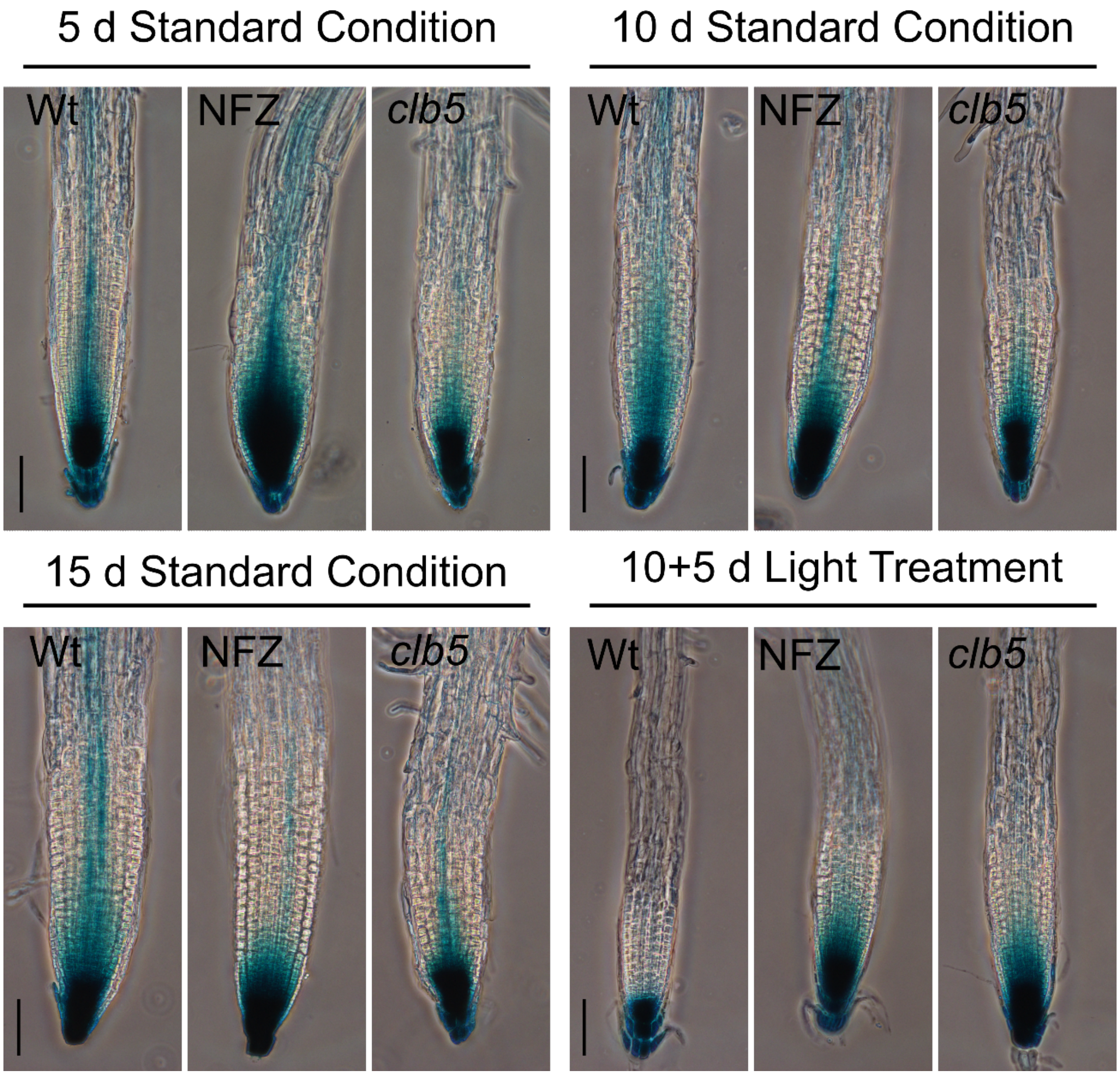

Supplementary Figure S8. Auxin response in the primary root in ACS1-accumulating seedlings. Auxin response *proDR5::GUS* marker of the primary root tip from Ler, Ler treated with norflurazon (NFZ), and *clb5* seedlings grown under standard light conditions (SLC; 100 μmol m^{-2} sec^{-1}) for 5, 10 and 15 days, or 10-day-old in standard light conditions (SLC) and 5 days under low light (LL; 10+5). Auxin maxima is detected in the RAM region in the root and the columella. Scale bar corresponds to 200 μm.

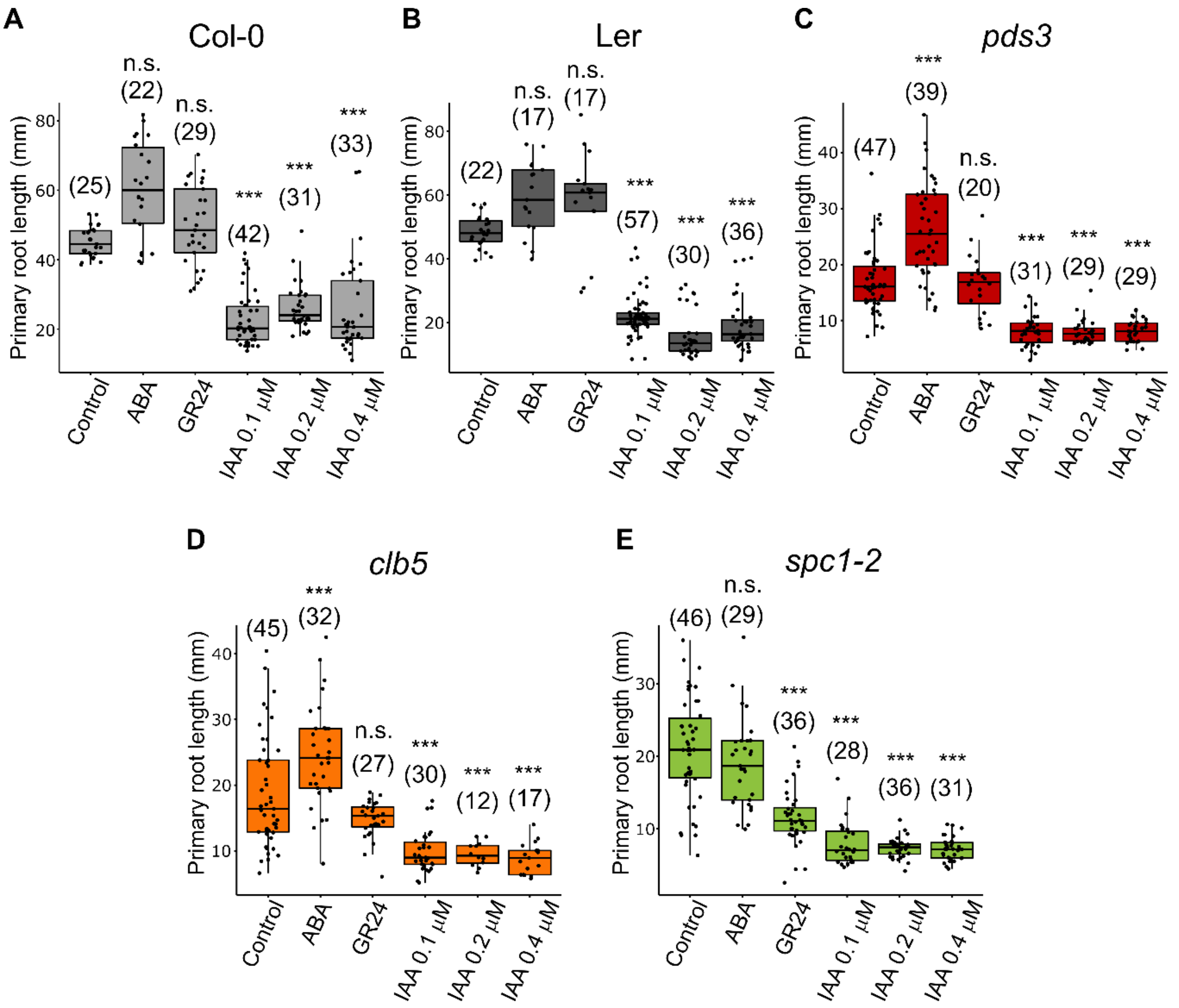

Supplementary Figure S9. Response of the primary root to hormonal treatments. Quantification of the primary root length in 15-day-old from Col-0 (A), and Ler (B) Wt seedlings, and carotenoid deficient *pds3* (C), *clb5* (D) and *spc1-2* (E) mutants. Plants were treated with 0.5 µM ABA, 0.4 µM GR24, 0.1, 0.2 and 0.4 µM auxin for 9 days under standard light conditions (SLC; 100 µmol m^{-2} sec^{-1}). Boxes represent the 25th and 75th percentiles, and the solid line indicates the median, and the whiskers denote the minimum and maximum values of each group. Asterisks indicate statistically significant differences relative to the corresponding Wt. Multiple comparison were performed using non-parametric one-way Kruskal-Wallis, and Dunn's test was used for pairwise comparison (α=0.005). n.s. denotes no statistically significant differences (P≥0.01). Numbers in parentheses indicate the total pooled individuals across three independent analyses.

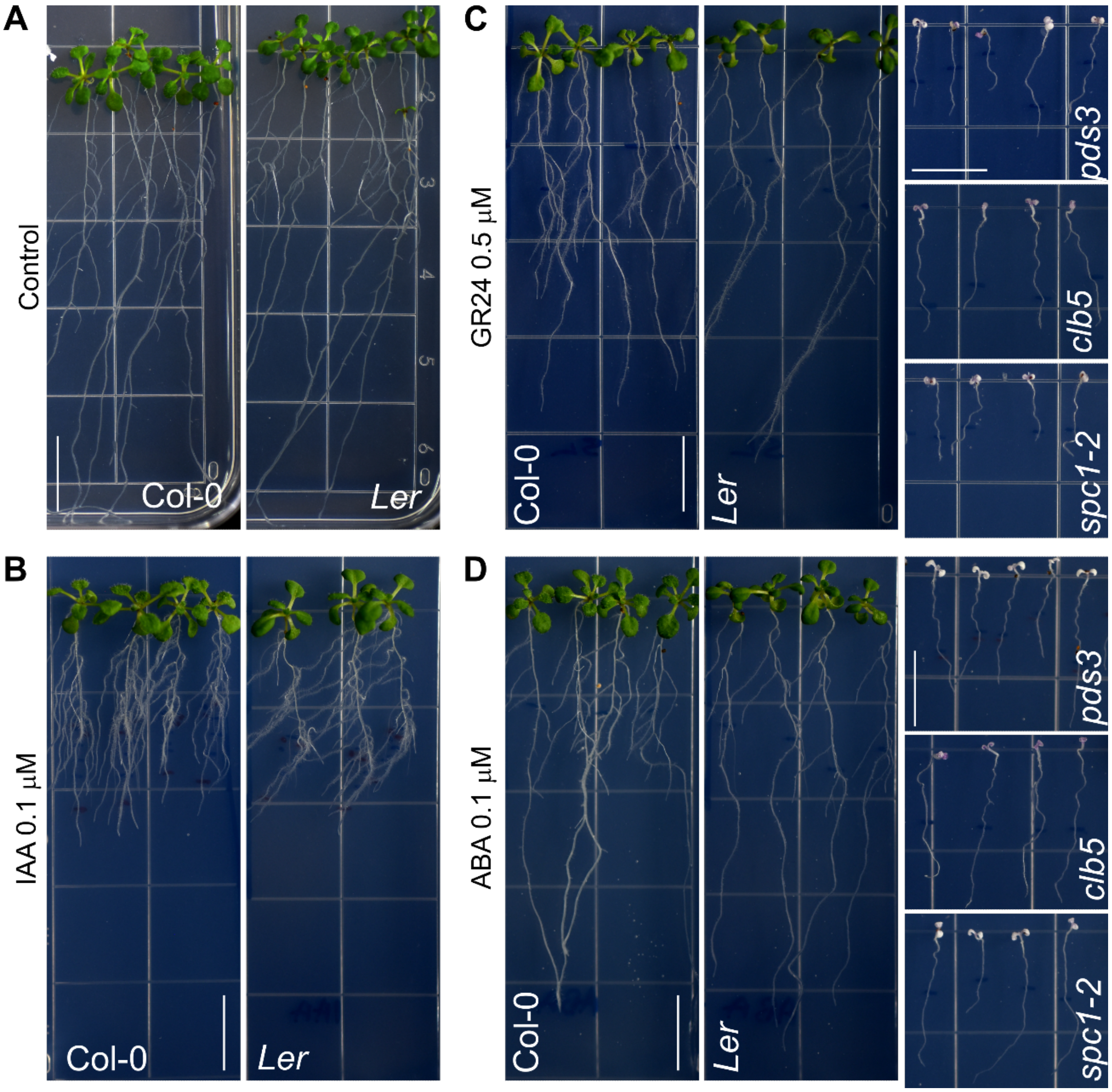

Supplementary Figure S10. **Effect of ABA and SL supplementation on lateral root development in ACS1-accumulating mutants.** Representative images of lateral root development of Col-0 and Ler Wt seedlings and carotenoid-deficient *pds3*, *clb5* and *spc1-2* mutants after being transferred to GM medium (A) or medium supplemented with 0.1µM IAA (B), 0.5 µM GR24 (C) and 0.1 µM ABA (D) for 9 days. Seedlings were grown in standard light conditions (SLC 100 µmol m^{-2} sec^{-1}) and lateral root number (LR) was analyzed in 15-day-old plants. Scale bar corresponds to 2 cm (scale shown in one image applies to all images in the panel).

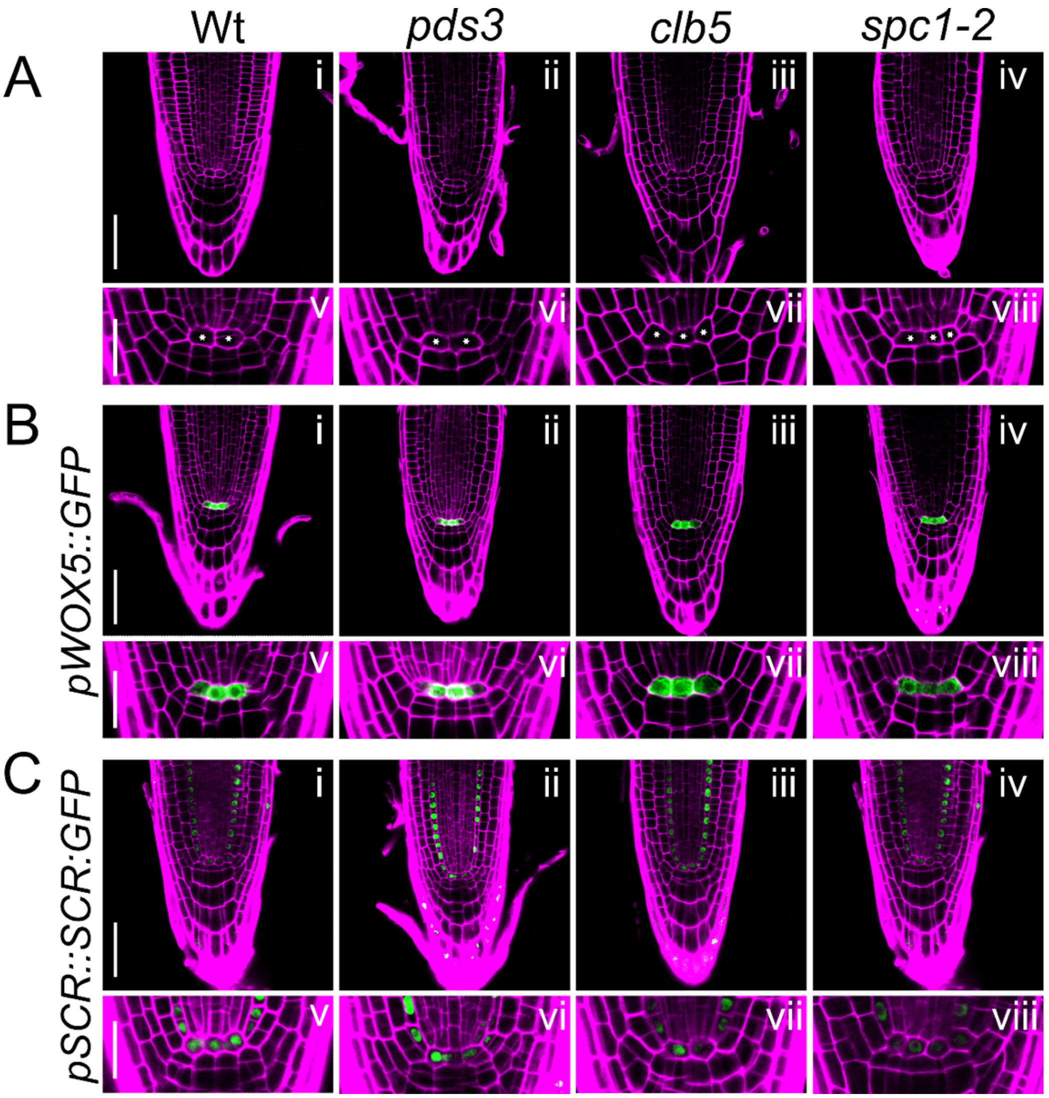

Supplementary Figure S11. Analysis of RAM and QC structure and expression QC markers. A) Representative images of RAM morphology in 5-day-old Col Wt (i), *pds3* (ii), *clb5* (iii) and *spc1-2* (iv) mutants and close-up views of the stem cell niche, including QC (v-viii). Asterisks indicate the QC cells. (B) GFP expression pattern of the *proWOX5::GFP* QC marker in the RAM (i-iv) and close-up views of the QC (v-viii) of Col Wt, *pds3*, *clb5* and *spc1-2* seedlings. (C) Expression pattern of endodermis marker *proSCR::SCRYFP* in the RAM (i-iv) and corresponding close-up views of the QC region (v-viii) in Col Wt(i), *pds3* (ii), *clb5* (iii) and *spc1-2* (iv) mutants. For optimal visualization, samples were stained with propidium iodine (GFP 488 nm, filter 505-525 nm). Scale bar for A-C corresponds to 50 μm and 20 μm for the close-up views, scale shown in one image applies to all images in the panels A, B and C and corresponding close-ups.

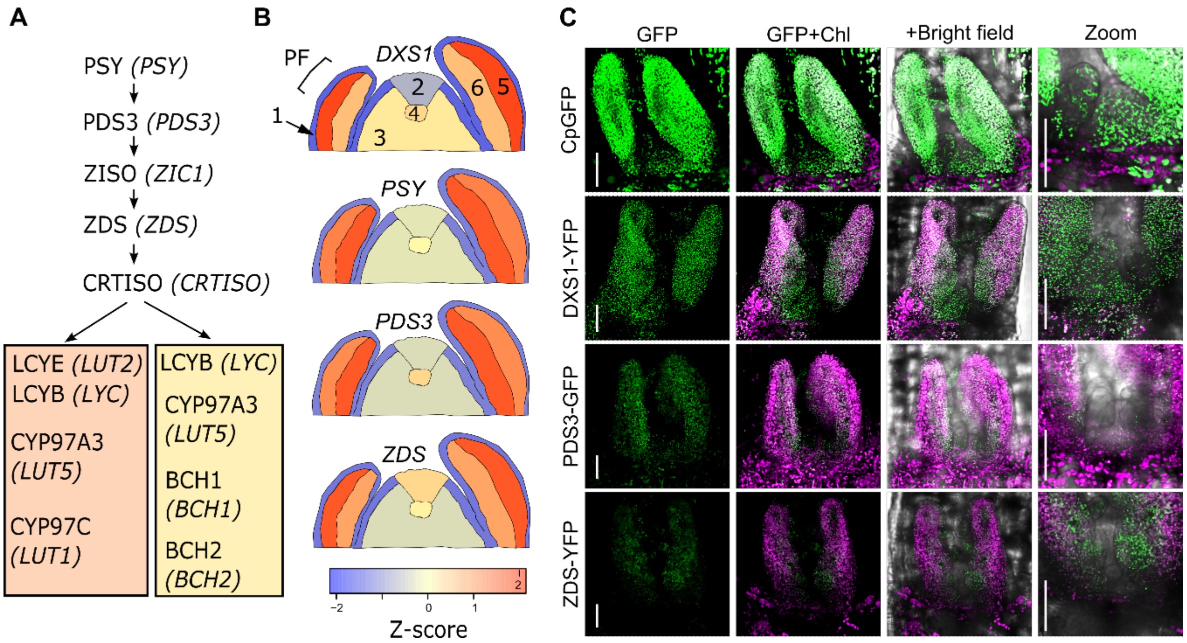

Supplementary Figure S12. Expression patterns in carotenogenic enzymes in the SAM. A) Diagram of genes encoding enzymes involved in carotenogenesis.

B) Expression patterns of the MEP and carotenogenic genes (*DXS1*, *PSY*, *PDS3* and *ZDS)*. The color scale represents the expression levels of the corresponding genes according to the Z-score shown in the heat map. C) Localization patterns of the plastid marker C-cpGFP, DXS1-YFP, PDS3-GFP and ZDS-YFP fusion proteins in the first pair of leaves and the shoot apical meristem (SAM) of 4-day-old Col Wt seedlings. The GFP and chlorophyll (Ch) signals are shown merged with the brightfield (BF) channel allow visualization of the organ structure. Scale bar for C corresponds to 50 μm, scale shown in one image applies to all images in the panels A, B and C and corresponding close-ups.

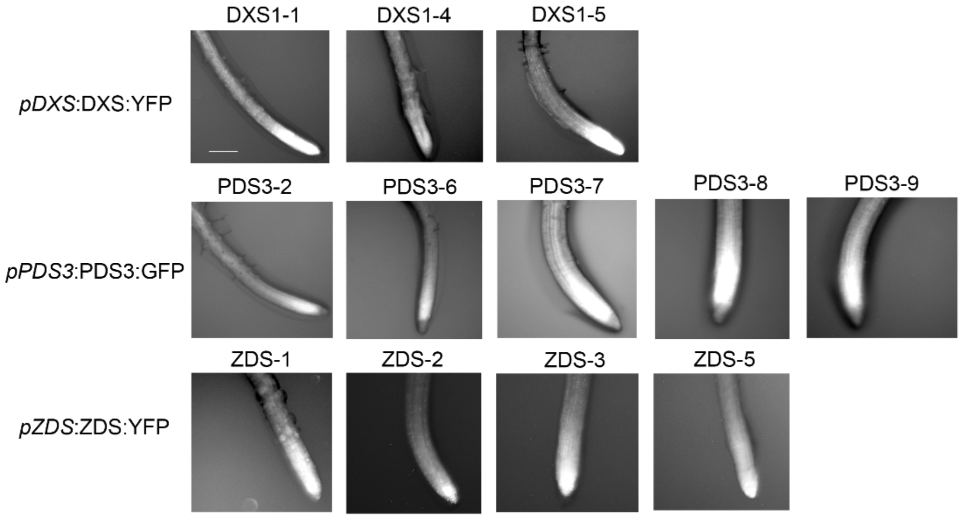

Supplementary Figure S13. Expression pattern of p*DXS1::DXS1:YFP,* p*PDS3::PDS3:GFP* and p*ZDS::ZDS:YFP* lines. Representative localization patterns of the DXS1-YFP, PDS3-GFP and ZDS-YFP fusion proteins in the primary roots of 10-day-old seedlings. Fluorescent signals were observed in independent transgenic lines using fluorescence microscopy. Images illustrate consistent expression patterns across lines. Scale bar corresponds to 250 µm (scale shown in DXS1-1 image applies to all images).

Supplementary Table S4. RAM length of Wild-type and albino mutants grown under standard light and low light conditions

Genotype	10 d SLC	15 d SLC	10+5 d LL	*P*-value[1]
Col-0	166.2 ± 10.6	151.65 ± 9.9	74.71 ± 16.9	<0.001
Ler	178.8 ± 17.0	169.60 ± 20.9	90.94 ± 16.7	<0.001
pds3	79.0 ± 15.2	104.97 ± 26.4	89.44 ± 26.4	0.008
clb5	84.4 ± 20.8	124.94 ± 20.2	102.50 ± 38.9	0.005
spc1-2	78.0 ± 9.9	102.29 ± 17.1	90.56 ± 10.7	<0.001
***P*-value[2]**	<0.001	<0.001	0.065	

[1]Kruskal-Wallis analysis by row.

[2]Kruskal-Wallis analysis by column.

Data show the RAM length average in mm and standard deviation. Standard light condition (SLC) and low light (LL) condition.

Supplementary Table S4. RAM length of Wild-type and albino mutants grown under standard light and low light conditions.

Supplementary Table S5. **Sequence of the oligonucleotides used in this study**

Name	Sequence 5′-3′
CLAp1800GW	CACCGAAGTCCGCGTACATTTTAGG
CLARNSTP	CAGAGCTTCCCTTGGTGCAC
PDS3BP Fw	AAAAAGCAGGCTTCGGAAGAGAAGACTGACAAGTC
PDS3nstp Rev	AGAAAGCTGGGTTTGATGATGATACTGTTGCCTCCG
ZDSFw	AAAAAGCAGGCTTCGAATGTGTGTGTGCCTACTACTG
ZDSRv	AGAAAGCTGGGTTGACGACTGAAGAAGCCATGGCC
TOC132 Fw	GAGGTTGAGGAAGGTGAAGG
TOC132 Rv	CCTTCACCTTCCACAACATCT
POL1B Fw	TGAATACCGTTCACGTGCCC
POL1B Rv	AGCCGCACTTCCCTGAACAG
ACT7 Fw	CAGTGTCTGGATCGGAGGAT
ACT7 Rv	TGAACAATCGATGGACCTGA
HEMA Fw	TCTATCTCCTTCTTCTTCTTCTT
HEMA Rv	CTCGTCTGGTTCTATTGTTCT
RPoTp Fw	TGGAAGCCGTCTGCTAGAACTA
RPoTp Rv	TGTCTGAATGCAGGTCGAAAC
RbcL Fw	GTGTTGGGTTCAAAGCTGGT
RbcL Rv	CATCGGTCCACACAGTTGTC
RpoA Fw	TTCTAACATAGCAGGTATTCAAG
RpoA Rv	GCGTTCCATATAGATTACTTCTT

Supplementary Table S6. Fixed-Effects two-way ANOVA for Root length for Wild type and carotenoid deficient mutants.

Predictor	Sum of Squares	*df*	Mean Square	*F*	*p*	partial η^2	partial η^2 90% CI [LL, UL]
(Intercept)	191.43	1	191.43	374.83	.000		
Treatment	51.91	5	10.38	20.33	.000	.10	[.07, .13]
Genotype	375.95	4	93.99	184.03	.000	.45	[.41, .48]
Treatment x Genotype	259.89	20	12.99	25.44	.000	.36	[.31, .39]
Error	457.10	895	0.51				

Note. LL and UL represent the lower-limit and upper-limit of the partial η^2 confidence interval, respectively.
